# Comprehensive analysis of antimicrobial resistance dynamics among broiler and duck intensive production systems

**DOI:** 10.1038/s41598-025-89432-z

**Published:** 2025-02-08

**Authors:** Zsombor Szoke, Peter Fauszt, Maja Mikolas, Peter David, Emese Szilagyi-Tolnai, Georgina Pesti-Asboth, Judit Rita Homoki, Ildiko Kovacs-Forgacs, Ferenc Gal, Laszlo Stundl, Levente Czegledi, Aniko Stagel, Sandor Biro, Judit Remenyik, Melinda Paholcsek

**Affiliations:** 1 Faculty of Agricultural and Food Sciences and Environmental Management, Complex Systems and Microbiome-innovations Centre, University of Debrecen, Debrecen, Hungary; 2 Faculty of Agricultural and Food Sciences and Environmental Management, University of Debrecen, Debrecen, Hungary; 3 Faculty of Agricultural and Food Sciences and Environmental Management, Institute of Animal Science, Biotechnology and Nature Conservation, Department of Animal Husbandry, University of Debrecen, Debrecen, Hungary; 4 Hungarian National Blood Transfusion Service Nucleic Acid Testing Laboratory, Budapest, Hungary; 5 Faculty of Medicine, Department of Human Genetics, University of Debrecen, Debrecen, Hungary

**Keywords:** Poultry resistome, Poultry microbiome, Prophylactic antibiotic use, Intensive broiler rearing, Intensive duck rearing, Biomarkers, Microbiology, Biomarkers

## Abstract

Antimicrobial resistance (AMR) is a global health challenge requiring cross-sector action, with research largely focused on chickens, leaving ducks underexplored. This study examines AMR dynamics in Ross 308 broilers and Cherry Valley ducks over 15 months and 15 stocking periods under consistent rearing conditions. A total of 96 pooled samples were collected: 50 from broiler farms (26 biological, 24 environmental) and 46 from duck farms (24 biological, 22 environmental). Using next-generation shotgun sequencing, 3,665 distinct AMR types were identified: 1,918 in broilers and 1,747 in ducks. Host-specific AMRs comprised 25.3% in broilers and 18% in ducks, while 56.7% were shared. AMR diversity declined across production phases, with broilers losing 641 types and ducks losing 308, yet AMR frequencies increased significantly by the finisher phase (*p* < 0.0001). Based on in silico data, prophylactic antibiotic use significantly reduced the prevalence of multidrug-resistant bacteria in both poultry species (*p* < 0.05). Hospital-acquired infection-associated AMRs were higher in broilers than in ducks at the start of production but declined significantly by the end of the rearing period (*p* < 0.0001). Above-average resistance markers accounted for approximately 10% of all detected resistance determinants. Tetracycline and phenicol resistances emerged as the most prevalent. 13 high-resistance carrier (HRC) species were shared between both hosts. Broiler-specific HRCs exhibited significantly higher abundances (relative frequency: 0.08) than duck-specific HRCs (relative frequency: 0.003, *p* = 0.035). The grower phase emerged as a critical intervention point. In farm environments 15 broiler-specific and 9 duck-specific biomarker species were identified, each strongly correlated with poultry-core HRCs (correlation coefficient > 0.7). Broiler exhibited higher abundances of key resistance genes, with tetracycline resistance predominantly associated with *Bacteroides coprosuis*, *Pasteurella multocida*, and *Acinetobacter baumannii*. Despite its limitations, this research provides key insights into AMR trends in two major poultry types, guiding targeted interventions and sustainable management strategies.

**Supplementary Information:**

The online version contains supplementary material available at 10.1038/s41598-025-89432-z.

## Introduction

Hungary accounts for approximately 3.5% of the European Union’s total poultry production, focusing on broiler chickens and ducks, making it a notable contributor to the EU poultry sector^1,2^. While its production volume is smaller compared to global leaders like the USA and China, Hungary plays an important role in meeting European poultry demand^3,4^.

As a major global source of animal protein, poultry farming plays a critical role in the dynamics of antimicrobial resistance (AMR)^5^.

Under intensive animal farming systems, antibiotic use plays a crucial role in preventing diseases and promoting growth performance. However, sub-therapeutic use of antibiotics is known to exert strong selection pressure, fostering the emergence and spread of antibiotic-resistant bacteria (ARBs)^5,6^.

As a result, ARBs and resistance genes are widespread in both poultry manure and farm environments threatening both public health and food safety^7,8^.

Beyond the above, horizontal gene transfer exacerbates the spread of resistance, turning beneficial microbiota, such as probiotics and short-chain fatty acid (SCFA) producers, into reservoirs for resistance genes^9^.

Foodborne pathogens and resistant bacteria are known to contaminate the environment, infiltrate the food chain, and pose significant public health risks^8^. To address the escalating threat of antimicrobial resistance, a comprehensive One Health approach that integrates human, animal, and environmental health is required^10^.

Research on intensive poultry farming has primarily focused on broilers, providing valuable insights into antibiotic use, resistance mechanisms, and AMR transmission through the food chain^11,12^. In contrast, research on ducks—a poultry sector of increasing economic and nutritional significance—remains limited and fragmented. Ducks are raised under distinct conditions, such as higher water usage, which fosters unique microbial ecosystems. These conditions likely drive differences in microbial community dynamics and resistance profiles when compared to broilers^13^, hence the need for studies that will explore resistance profiles among ducks’ production systems.

Furthermore, research on poultry AMR predominantly targets specific resistance-carrying species, resistance genes, or antibiotic classes, often overlooking a comprehensive evaluation of AMR diversity, abundance, and microbial interactions. Research predominantly focuses on pathogenic bacteria such as *Escherichia coli*,* Campylobacter*, and *Salmonella* due to their roles as foodborne pathogens and direct impacts on human health^14–16^. This limited scope, combined with a lack of comparative studies between broiler and duck production systems, restricts our understanding of species-specific AMR patterns and their broader public health implications.

Investigating how rearing practices influence resistance profiles across poultry species is crucial for developing more effective, tailored mitigation strategies.

Another significant gap in the current literature is the lack of system-wide analyses of AMR dynamics throughout the entire poultry production cycle. Additionally, while the transmission of foodborne pathogens from poultry to humans is well-documented, the role of poultry farming systems in harboring resistance genes linked to healthcare-associated infections (HAIs) remains underexplored^17^.

To address these challenges, a more holistic approach to AMR research is needed—one that includes commensal bacteria and their interactions with pathogens, as well as comprehensive analyses of resistance dynamics across diverse production systems. Such efforts would provide critical insights for developing robust, tailored strategies to mitigate AMR risks and safeguard both public and environmental health.

## Results

### Description of the study

**Fig. 1:**
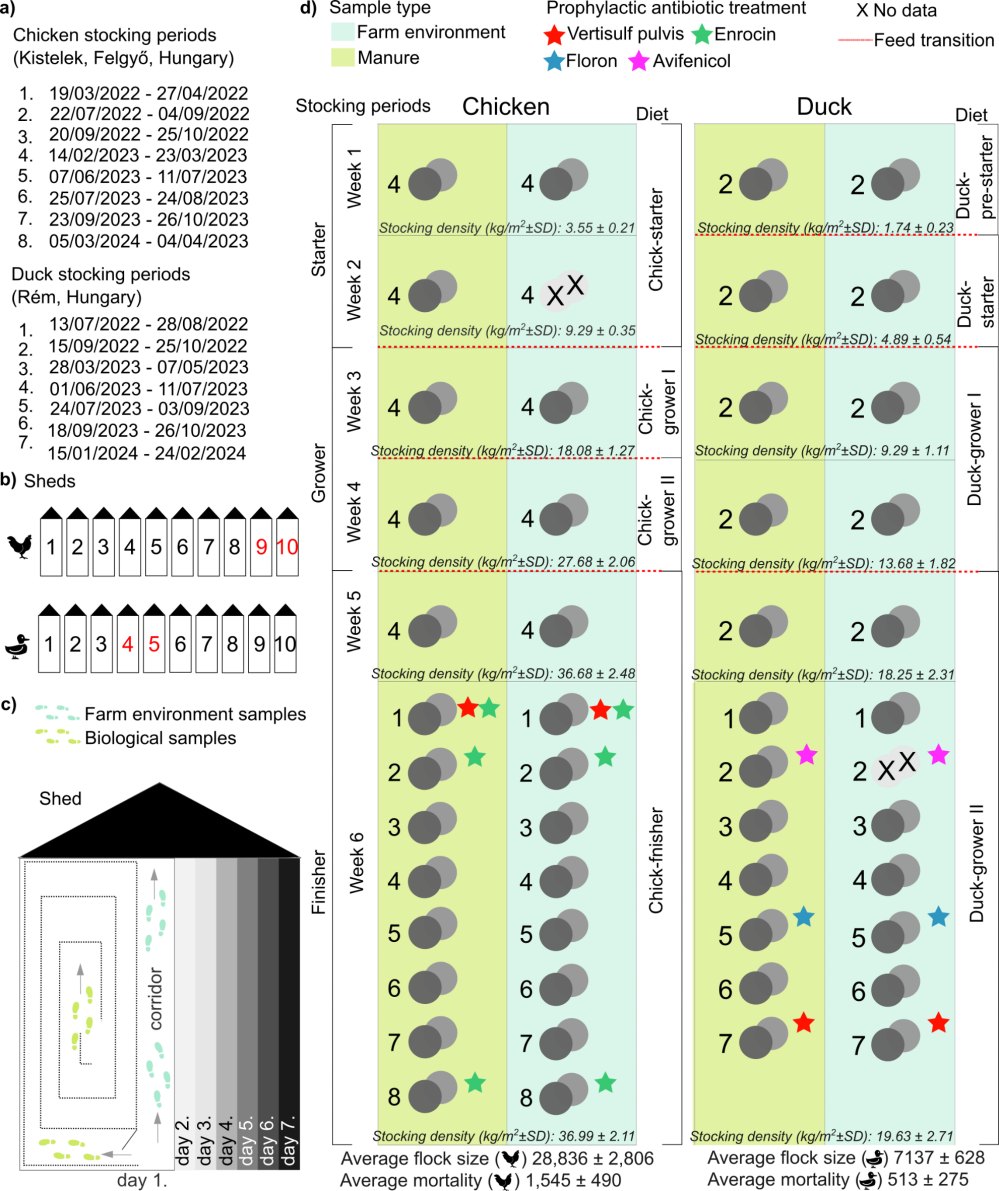
Overview of the study. (**a**) The study was conducted between March 2022 and April 2024 at two locations—Kistelek, Hungary and Felgyő, Hungary for broilers, and Rém, Hungary for ducks. During this period, there were 8 stocking cycles for broiler chickens and 7 stocking cycles for ducks. (**b**) The figure highlights the specific sheds used for the experiment. For broiler chickens, Sheds 9 and 10 were utilized to allow biological replicates, while for ducks, Sheds 4 and 5 were selected for the same purpose. (**c**) The figure also illustrates the origin of the sample pools and the process for creating them, including the sources of biological and environmental samples and their collection schedule. Environmental footpad samples were taken daily from the walkways inside the sheds, while biological footpad samples (feces) were collected from the areas where the birds were housed. These collections were performed every day of the week (Day 1 to Day 7) and combined to represent a single biological or environmental sample pool. Both species were housed in temperature-controlled environments and managed according to strict biosecurity and vaccination protocols. (**d**) Sampling covered the starter, grower, and finisher phases, standardized into three 2-week stages for both broilers and ducks. Birds were fed species-specific diets formulated in-house by the production company to meet their nutritional requirements: ducks were fed ‘Duck-pre-starter’, ‘Duck-starter’, ‘Duck-grower I’, and ‘Duck-grower II’ feeds, while chickens received ‘Chick-starter’, ‘Chick-grower I’, ‘Chick-grower II’, and ‘Chick-finisher’ feeds. Feed transitions are marked with red lines in the figure, and weekly changes in stocking density (kg/m²) are detailed. Flock sizes for both broilers and ducks are indicated, and prophylactic antibiotic treatments, such as Vertisulf Pulvis (red), Enrocin (green), Floron (blue), and Avifenicol (purple), are marked with stars.

Our study focused on poultry raised under intensive farming conditions, specifically the most commonly farmed species in Hungary: Ross 308 broiler chickens (Kistelek and Felgyő, Hungary) and Cherry Valley ducks (Rém, Hungary). Sampling was conducted between March 19 and April 27, 2022, and March 5 to April 4, 2024. The company responsible for broiler chicken and duck meat production, Hungerit Zrt., operates its own breeding stock and hatchery. This ensures that day-old poultry (both ducks and chickens) are bred in-house following established protocols and delivered to farms under strictly controlled conditions. Both broilers and ducks were housed in temperature-controlled environments under standard rearing conditions and managed according to industry-standard practices, including vaccination schedules and biosecurity measures to minimize disease risks. The broiler flocks averaged 28,836 birds ± 2806, while duck flocks averaged 7,137 birds ± 628. Mortality rates were negligible, recorded at an average of 1545 birds ± 490 for broilers and 513 birds ± 275 for ducks. To ensure a precise comparison of production systems, both were standardized into three production stages—starter, grower, and finisher—each spanning two weeks. Ducks underwent a feed transition within the starter phase, moving from ‘Duck-pre-starter’ to ‘Duck-starter’ feed after one week, followed by ‘Duck-grower I’ and ‘Duck-grower II’ feeds during the grower and finisher stages, respectively. Broilers, in contrast, were fed ‘Chick-starter’ feed throughout the starter phase, transitioning between ‘Chick-grower I’ and ‘Chick-grower II’ feeds during the grower stage, and receiving ‘Chick-finisher’ feed exclusively in the final phase. Over the 15-month study spanning 15 production cycles, one broiler chicken cycle (No. 4: 14/02/2023–23/03/2023) and one duck cycle (No. 2: 15/09/2022–25/10/2022) were monitored daily throughout their production phases. In the remaining cycles, pooled samples from the final week, representing critical pre-slaughter conditions, were analyzed. Prophylactic antibiotic treatments were used in 3 of 8 broiler cycles and 3 of 7 duck cycles. Ducks received Vertisulf Pulvis and Enrocin (sulfonamides, diaminopyrimidines, fluoroquinolones), while broilers were treated with Vertisulf Pulvis, Floron, and Avifenicol (sulfonamides, diaminopyrimidines, fluoroquinolons, and phenicols). Samples were collected across all rearing phases—starter, grower, and finisher. In broilers, stocking density increased from 3.55 kg/m^2^ in the starter phase to 36.99 kg/m^2^ in the finisher phase, a tenfold increase. For ducks, density rose from 1.74 kg/m^2^ to 19.63 kg/m^2^, approximately an elevenfold increase. Biological (manure) and environmental (farmhouse) samples were collected daily and pooled weekly, with each pool consisting of seven representative subsamples collected every week from Monday to Sunday. A total of 96 pooled samples were collected: 50 from broiler farms (26 manure, 24 farmhouse) and 46 from duck farms (24 manure, 22 farmhouse). Sampling occurred at six time points (1, 2, 3, 4, 5, and 6 weeks of age) across the production cycle, with biological and environmental samples collected simultaneously (Fig. 1).

### Description of the shotgun sequencing results

Shotgun metagenomic sequencing was carried out on the Illumina NovaSeq platform (Illumina, USA). A total of 42,496,757 ± 7,338,415 reads per sample were obtained in duck, and 46,493,771 ± 8,826,263 in chicken samples. The average number of reads of *Archaea* was (duck: 1,062 ± 1,249; chicken: 1,084 ± 3,444), *Bacteria* was (duck: 42,229,138 ± 7,418,026; chicken: 45,799,522 ± 8,837,808), *Eukaryota* was (duck: 163,709 ± 406,924; chicken: 492,977 ± 1,047,765), and *Virus* was (duck: 102,848 ± 191,455; chicken: 200,188 ± 327,869) reads per sample.

### Comparative analysis of AMR types, abundance, and associated species diversity across rearing phases in broilers and ducks

We aimed to compare AMR dynamics in broiler and duck-rearing systems by evaluating cumulative AMR abundance, diversity, and species composition across production phases to identify host species-specific resistance patterns and trends.

**Fig. 2:**
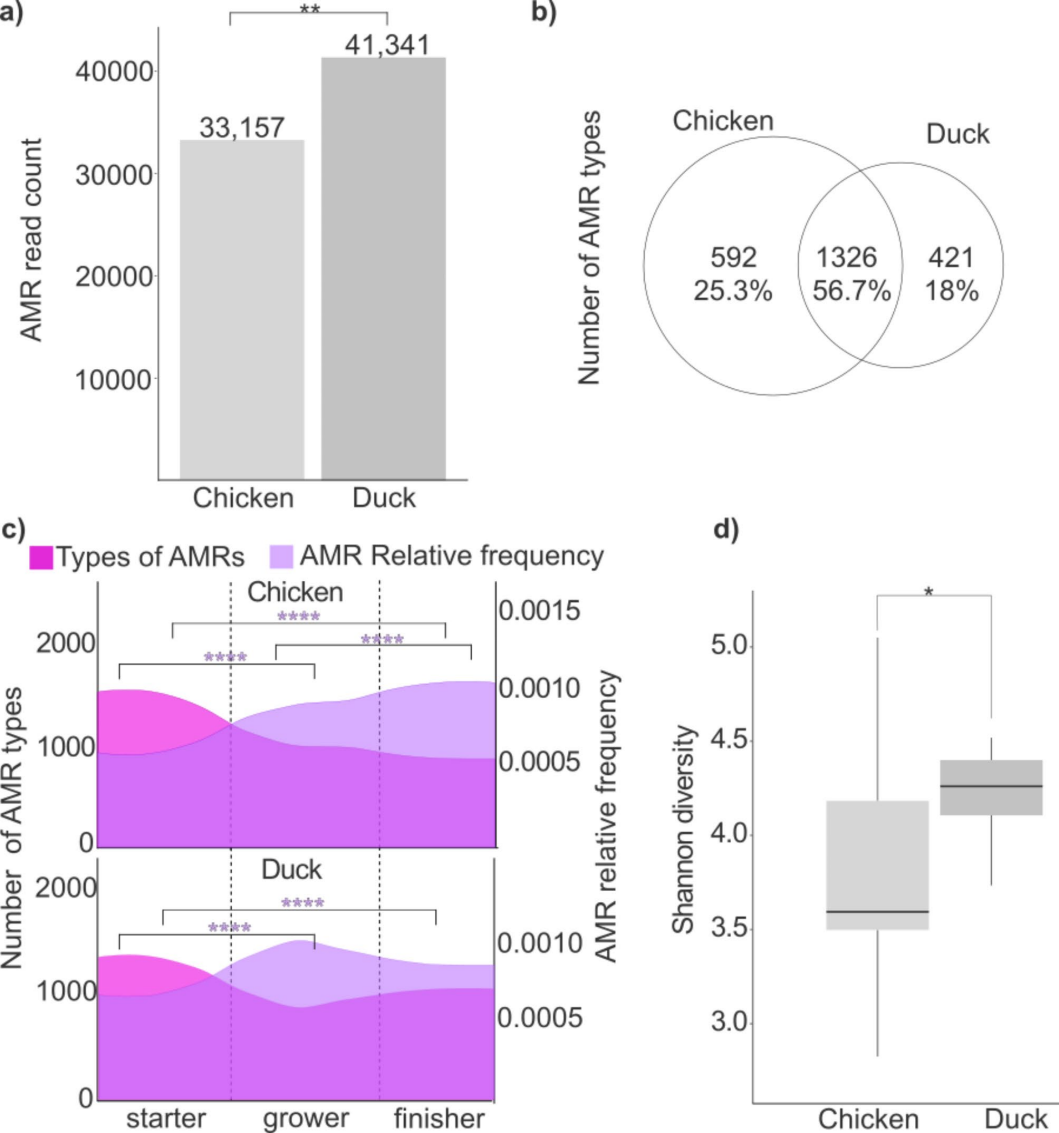
Comparative analysis of antimicrobial resistance profiles: AMR types, relative frequencies, and associated taxonomy in chickens and ducks across rearing phases. (**a**) Cumulative AMR read counts in manure samples from broilers and ducks collected throughout the entire rearing period. (**b**) Comparison of the number of AMR types between broilers and ducks, showing shared and unique AMRs for each species. (**c**) Dynamics of AMR diversity (y1-axis) and relative frequency (y2-axis) across the starter, grower, and finisher phases and between the two species. (**d**) Taxonomic diversity of AMR-carrying species, represented by the Shannon index. Asterisks indicate significant difference (*p < 0.05, **p < 0.01, ****p < 0.0001).

The cumulative number of antimicrobial resistance genes in manure samples from broilers and ducks was assessed by analysing AMR read count data from systematically collected samples over the entire rearing period (Fig. 2a). Overall, resistance levels in duck samples proved to be significantly higher than in chicken samples (duck AMR read count: 41,431; chicken AMR read count: 33,157; *p* = 0.0073).

The core antimicrobial resistance types were analyzed for both bird species, along with the distinct host-specific AMR types unique to broiler chickens and ducks. Venn diagrams illustrate the shared core and unique host-specific AMR occurrences for each species (Fig. 2b), while the Supplementary File 1 provides a detailed breakdown of the resistance types that were either shared or uniquely associated with each host bird (Fig. 2b). Overall, the spectrum of AMR varieties was very similar for broilers and ducks, with 1,918 AMR varieties identified in chickens and 1,747 in ducks. The shared AMR varieties between broilers and ducks accounted for 56.7% (1,326 out of 2,339) of all detected AMRs. Chicken manure samples contained more unique, host-specific AMR arrays than ducks, with broilers exhibiting 592 AMR types (25.3%) and ducks showing 421 unique types (18%).

The study further investigated changes in the number of AMR types and their corresponding relative frequencies across the three rearing stages—starter, grower, and finisher—for chickens and ducks (Fig. 2c). Notably, opposite trends were observed between the range of antimicrobial resistances and their associated cumulative relative frequency values for both host species.

The spectrum of the AMRs was highest during the starter phase for both species, 1543 in broilers and 1416 in ducks. Broilers showed a continuous decline in AMR arrays throughout the rearing period. In ducks, however, the number of AMR types decreased only until the grower phase—where it reached the lowest level of the entire rearing period (number of AMR types: 974) before showing a slight increase in the finisher phase (number of AMR types: 1108). Although the number of AMR varieties decreased in both bird species by the end of the finisher phase, the reduction from starter to finisher was significantly more pronounced in chickens (broilers: 641 AMR varieties vs. ducks: 308) For both broiler and duck, differences between starter and finisher phases were significant *p* < 0.0001.

The trends in the relative frequency of AMRs were inversely mirrored by the changes in the number of AMR types observed, highlighting an opposing relationship between AMR abundance and diversity. In both bird species studied, significantly higher relative frequency values for AMR were measured during the finisher phase compared to the starter phase. For broilers, the values for the starter and finisher phases were statistically significant (broiler starter AMR relative frequency: 0.0006 vs. broiler finisher: 0.0011, *p* < 0.0001), and for ducks as well (duck starter: 0.0007 vs. duck finisher: 0.0009, *p* < 0.0001). In chickens, there was a continuous and significant increase in AMR relative frequencies throughout the entire rearing period. However, this pattern was not observed in ducks. In ducks, the highest AMR relative frequency was detected during the grower phase, which was significantly higher than in the starter phase (starter AMR relative frequency: 0.0007, grower: 0.001, *p* < 0.0001). A noticeable decrease followed this in the finisher phase (finisher AMR relative frequency: 0.0009). Overall, AMRs were more abundant at the end of the finisher phase compared to the starter phase for both species, with chickens consistently showing higher AMR abundances than ducks by the finisher phase (chicken finisher AMR relative frequency: 0.0011 vs. duck: 0.0009, *p* = 0.39). These findings highlight the distinct dynamics of AMR development across different rearing phases and species, emphasizing the influence of host-specific factors and environmental conditions.

In silico identification of potential AMR-carrying taxa, based on taxonomic data, revealed that ducks exhibited a significantly greater Shannon alpha diversity (Shannon index: ducks 4.30 ± 0.37 vs. broilers 3.85 ± 0.57, *p* = 0.0011) (Fig. 2d).

### The impact of prophylactic antibiotic use on multidrug-resistant bacteria

We aimed to evaluate the impact of prophylactic antibiotic use on multidrug-resistant bacteria (MDRBs) in broiler and duck production systems by comparing AB-clean (no antibiotics) and AB-treat (with antibiotics) cycles by recording prophylactic antibiotic use in 37.5% of broiler cycles (3/8) and 42.9% of duck cycles (3/7).

MDRBs were identified in silico through the comprehensive analysis of resistance and taxonomic data derived from shotgun-sequenced metagenomes.

**Fig. 3:**
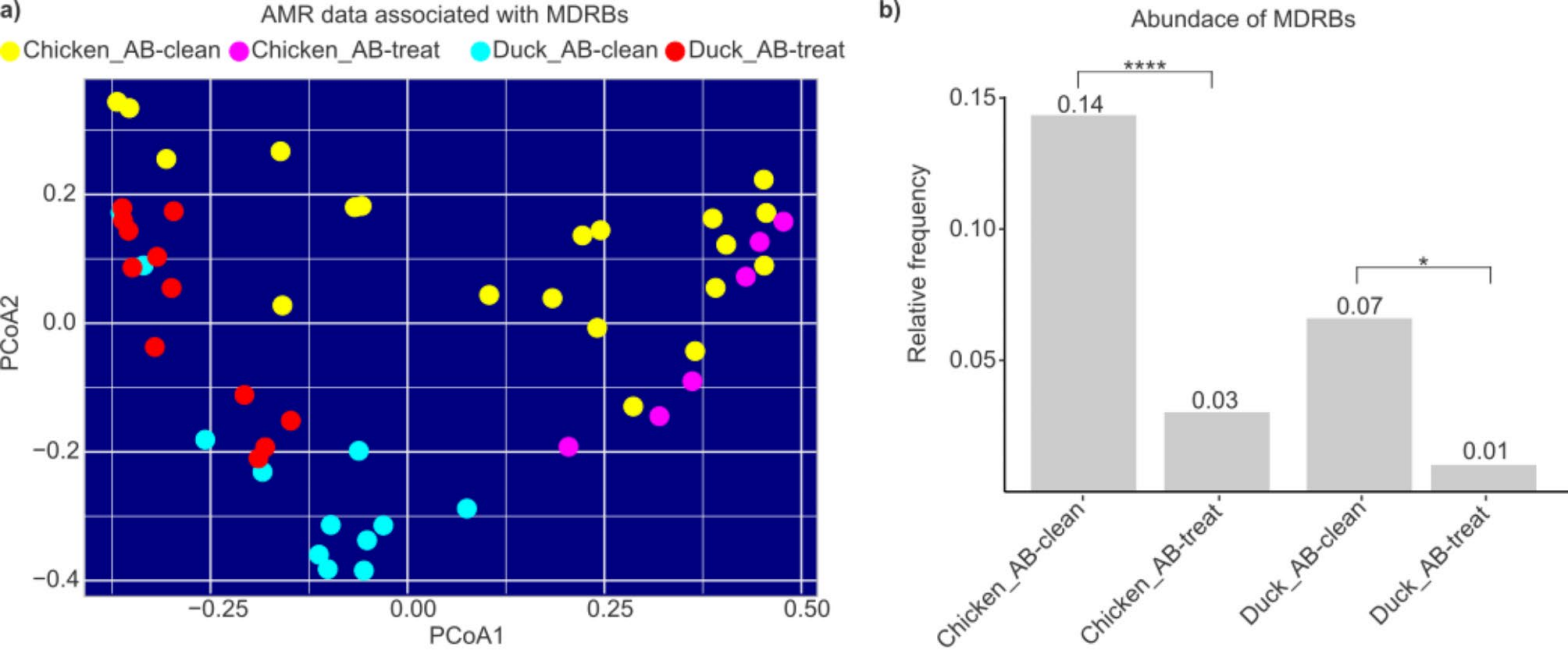
Impact of antibiotic treatments on MDRB abundance and resistance patterns in broiler chicken and duck production systems. (**a**) Principal coordinate analysis (PCoA) plot showing the distribution patterns of antimicrobial resistance (AMR) data associated with multidrug-resistant bacteria (MDRBs) in broiler chicken and duck samples. (**b**) Comparing the relative frequency data of MDRBs in broiler chicken and duck production systems revealed notable differences based on antibiotic treatment conditions. Groups were categorized as ‘AB-treat’ (those receiving any antibiotic treatment) and ‘AB-clean’ (those without treatment), allowing for a detailed assessment of how antibiotic use influenced MDRB prevalence across the two production systems. Asterisks indicate significant differences (****p < 0.0001, *p < 0.05).

The distribution patterns of samples based on their AMR data associated with MDRBs in broiler chicken and duck samples showed no notable alterations between the AB-clean and AB-treated sample populations (Fig. 3a).

Then the abundances of MDRBs in our different sample groups were further analysed and it was demonstrated that in production cycles with prophylactic antibiotic use, the abundance of multidrug-resistant bacteria was significantly reduced compared to AB-clean cycles (Fig. 3b). This reduction was particularly notable in broilers, where the relative frequency of MDRBs decreased from 0.14 in AB-clean cycles to 0.03 in antibiotic-treated cycles (*p* < 0.0001). Similarly, in ducks, the relative frequency of MDRBs was reduced from 0.07 in AB-clean cycles to 0.01 in antibiotic-treated cycles (*p*-value: 0.013).

### Rearing-specific variations in the AMR assortment, abundance, and carrier frequency among foodborne pathogens, HAIs, and commensals

Following the identification of AMRs, an in silico analysis was conducted to associate these resistances with bacterial species, specifically targeting foodborne pathogens, healthcare-associated infection-causing organisms, and commensal species, including short-chain fatty acid producers and probiotic strains (Supplementary File 2).

**Fig. 4:**
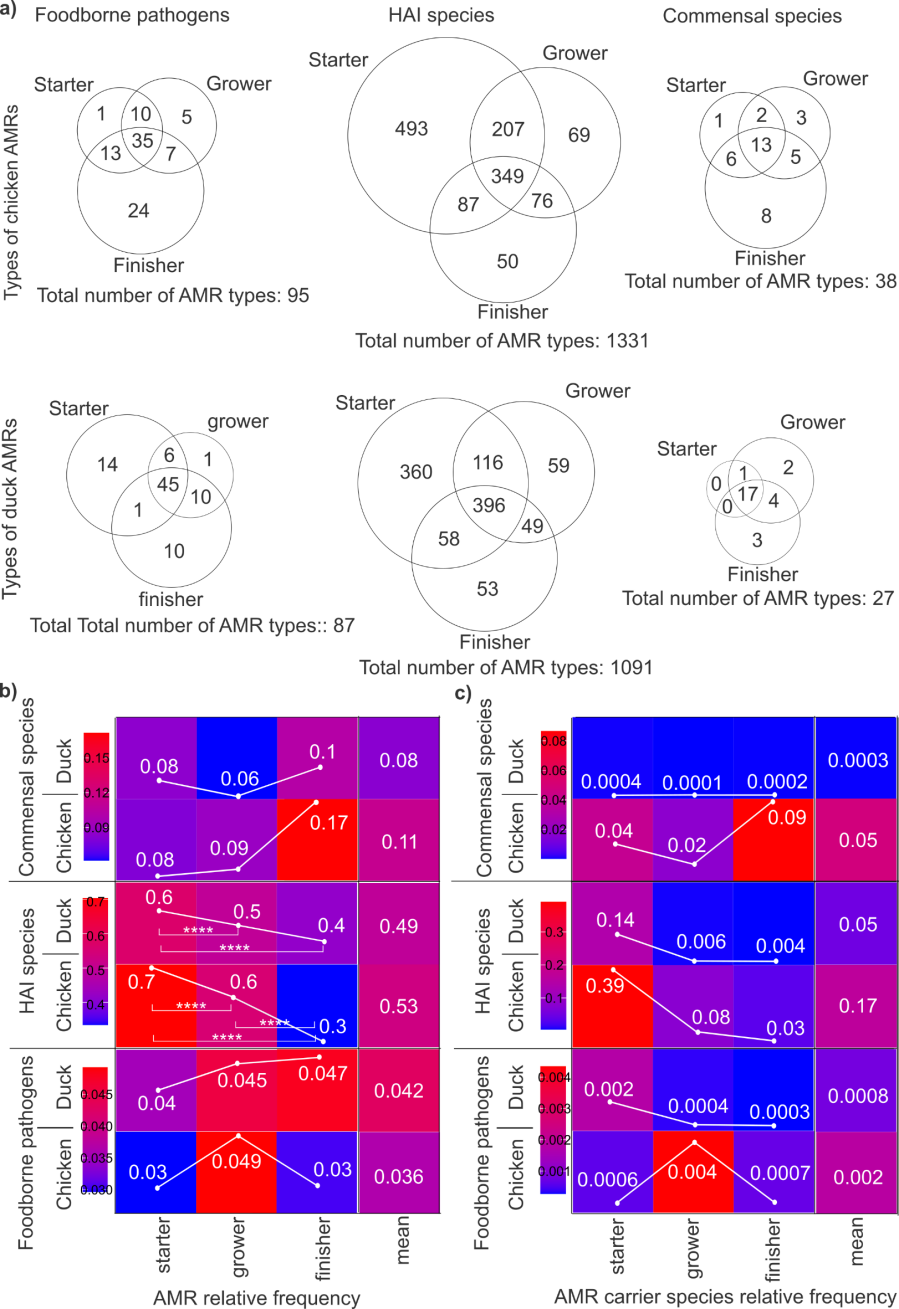
Rearing-specific variations in the AMR assortment, abundance, and carrier frequency among foodborne pathogens, HAIs, and commensals. (**a**) Venn diagrams illustrate the distribution of AMR types associated with foodborne pathogens, healthcare-associated infection (HAI)-causing organisms, and short-chain fatty acid (SCFA) producers across rearing phases (starter, grower, finisher) in both chicken and duck production systems. (**b**) Heatmaps, and line plots depict the relative frequency changes of AMRs associated with the designated bacterial groups (foodborne pathogens, HAIs, commensal species) across rearing phases in chickens and ducks. (**c**) Heatmaps, and line plots displaying rearing-specific changes in the relative frequencies of AMR carrier species across the three bacterial groups highlight more pronounced differences in broilers than in ducks. Asterisks indicate significant difference (****p < 0.0001).

The AMRs associated with these groups of species were analysed, with particular emphasis on the distribution of their various types across the rearing phases (starter, grower, and finisher) in both chicken and duck production systems (Fig. 4a).

Broilers exhibited a broader spectrum of antimicrobial resistance types compared to ducks. A total of 1,464 distinct AMR types were identified in broilers, while ducks showed 1,205 types. Among these AMRs, the majority were linked to healthcare-associated infection causing pathogens (HAIs), with 1,331 types found in broilers and 1,091 in ducks. Following this, AMRs associated with foodborne pathogens comprised 95 types in broilers and 87 in ducks. In broilers, the number of AMR types associated with foodborne pathogens steadily increased throughout the rearing period, starting with 1 type unique in the starter phase, rising fivefold in the grower phase, and reaching 24 types unique in the finisher phase. In contrast, ducks displayed a different pattern: the highest diversity of foodborne pathogen-associated AMRs was observed in the starter phase with 14 unique types, then dropped sharply to just 1 type in the grower phase, before rising again to 10 unique types in the finisher phase. Reassuringly, the array of AMRs associated with HAIs showed a decreasing trend in both chickens and ducks, dropping from 493 to 50 unique in broilers and from 360 to 53 unique in ducks between the starter and finisher phases. A notable number of AMRs were also linked to commensals, with the diversity of these resistances increasing over the production cycles in both bird species—38 different types in chickens and 27 in ducks.

The study further examined changes in the relative frequency of AMRs potentially associated with designated bacterial groups, including foodborne pathogens, HAI causing species, and commensal species, across the rearing phases (Fig. 4b). No significant changes were observed in the frequencies of AMRs associated with foodborne pathogens and commensal species throughout the rearing period. However, for HAIs, a significant decrease was detected, with substantially fewer HAI-associated AMRs observed in the finisher phase compared to the starter phase. In broilers, the HAI species relative frequencies declined significantly throughout the entire rearing period, from 0.7 in the starter phase to 0.3 in the finisher phase (*p* < 0.0001). In ducks, however, the reduction was more moderate, decreasing from 0.6 in the starter phase to 0.4 in the finisher phase (*p* < 0.0001), with a significant decrease observed between the starter and finisher phases, and also between the starter and grower phases (starter relative frequency: 0.6, grower relative frequency: 0.5, *p* < 0.0001). There was no significant difference between rearing periods in both broilers and ducks regarding AMRs linked to commensal species. A modest increase was observed, with frequencies rising from 0.08 to 0.17 in chickens, and from 0.08 to 0.1 in ducks. Regarding rearing-specific average AMR relative frequency values, there was no significant difference between chicken and duck systems. However, slightly more AMRs associated with commensal species and HAIs were detected in chickens compared to ducks (commensal species relative frequency: chicken 0.11 vs. duck 0.08; HAIs relative frequency: chicken 0.53 vs. duck 0.49).

When analysing the rearing-specific changes in the relative frequency of AMR-associated species, more pronounced differences were observed in broilers compared to ducks across all three microbial groups (Fig. 4c). Notably, a substantial 167-fold increase in relative frequencies was detected for commensal species in broiler-rearing systems, compared to duck-rearing systems (duck commensal species relative frequency: 0.0003, chicken: 0.05) highlighting a remarkable shift in their prevalence. Based on average relative frequency data, broilers exhibited 3.4 times more species associated with HAI species (HAI species relative frequency in ducks: 0.05, chicken: 0.17) and 2.5 times more foodborne pathogens potentially linked to the detected AMRs compared to ducks (foodborne pathogens relative frequency duck: 0.0008, chicken: 0.002).

### Comparative analysis of high-resistance carrier species across rearing phases in broiler and duck systems

Our objective was to identify the bacterial species classified as high-resistance carriers (HRCs), which harbor the highest number of resistance determinants, based on both biological and environmental samples from broiler and duck production systems. This analysis also considered the production stages—starter, grower, or finisher—to determine during which phase these HRCs were most prevalent. We further aimed to distinguish HRC species that were unique to either broiler or duck systems from those that were shared across both.

**Fig. 5:**
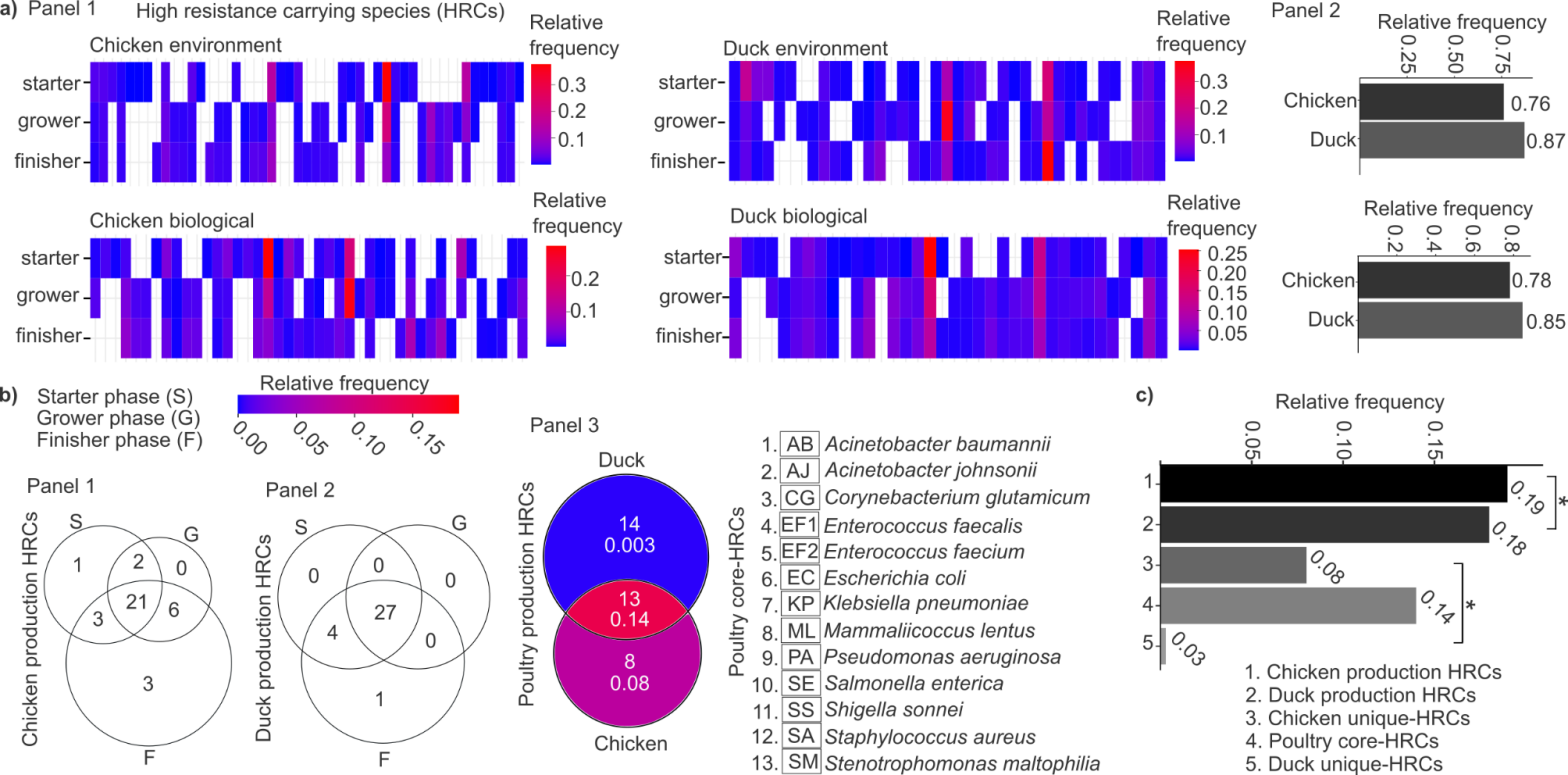
Identification of High-Resistance Carrier species (HRCs) across chicken and duck rearing phases: core-, unique HRCs, associated AMR patterns, and taxonomic insights. (**a**) Heatmaps depict the relative frequencies of the AMRs linked to the top 30 HRC species across the three rearing phases—starter, grower, and finisher—in environmental and biological samples from broiler chicken and duck production systems. Columns represent individual HRC species, with species names and associated antimicrobial resistance relative frequencies provided in Supplementary File 1. Barplots show the cumulative relative frequency of AMR abundances associated with HRC species in environmental and biological samples. (**b**) Value-weighted Venn diagrams illustrate the number of uniquely present and overlapping core HRC species across rearing phases. Corresponding heatmaps highlight the cumulative relative frequencies of HRC species within each Venn diagram segment. (**c**) Bar plots display the cumulative relative frequencies of core HRC species across five categories: chicken production HRCs, duck production HRCs, poultry core HRCs shared by both hosts and unique core HRC species found exclusively in chickens or ducks. Asterisks indicate significant difference (* *p* < 0.05).

Heatmaps depict the relative frequencies of the top 30 high-resistance carriers, each potentially linked to the highest antimicrobial resistance burdens, across the three rearing phases—starter, grower, and finisher—in both environmental and animal samples from broiler chicken and duck production systems (Fig. 5a, **Panel 1**). In our analysis of the AMR abundances associated with these HRC species, we found similar trends in both environmental and biological samples. In the manure, the cumulative AMR frequency was lower for broilers, at 0.78, compared to ducks, which had a cumulative AMR abundance of 0.85. Similarly, in the environmental samples, broilers had a cumulative AMR frequency of 0.76, while ducks showed a higher cumulative AMR abundance of 0.87. In both scenarios, ducks exhibited greater cumulative AMR abundance values within their respective HRC groups (Fig. 5a, **Panel 2**).

Columns of the heatmaps corresponded to specific HRC species, with their names and associated AMR data detailed in Supplementary File 3. Several HRC species were consistently detected across all feeding phases. In the chicken farm environment, 13 out of 49 species (26.5%) were consistently present. In chicken biological samples, 16 out of 43 species (37.2%) showed continuity across phases. In duck environmental samples, 20 out of 39 species (51.3%) were consistently detected, while in duck biological samples, 22 out of 36 species (61.1%) demonstrated persistence throughout the rearing process. These findings indicate a higher proportion of persistent HRC species in ducks compared to chickens, especially in biological samples. Based on these HRCs, we identified an HRC species pool for chicken and duck (species pool for chicken: 43 HRC species, species pool for duck: 36 HRC species) based on the species that were associated with specific rearing stages according to the highest resistance values.

Next, we aimed to identify the presence of HRCs consistently present throughout the production cycle, as well as those uniquely detected within specific rearing phases (Fig. 5b). Value-weighted Venn diagrams were created to represent the number of HRC species types across individual rearing phases, emphasizing their intersections.

Notably, no uniquely appearing HRC species were found in duck samples except in the finisher phase, where one species appeared exclusively (Fig. 5b, **Panel 2**). In contrast, three unique HRC species were identified in the broiler finisher phase. Additionally, six HRC species were commonly present in the intersection of the grower and finisher phases for broilers.

Across all three phases, 21 chicken core HRC species were shared in broiler samples, while 27 duck core HRC species were consistently present in duck samples throughout their rearing process (Fig. 5b, **Panel 1 and 2**).

We further investigated the core HRC species consistently found in chicken and duck samples and heatmaps were used to visualize the cumulative relative abundances associated with HRC species within each section (Fig. 5b, **Panel 3**). Our analysis identified 13 species consistently present across all rearing phases in both bird species, indicating a shared poultry core microbiome (poultry core HRCs) with significant resistance potential. Additionally, 14 HRC species were unique to duck, while 8 species were exclusive to chicken rearing system.

Based on the abundance data of the HRC species pools of both chicken, and duck, our taxonomic analysis revealed significant differences when comparing the relative frequencies of broiler HRC species with those of duck HRC species (chicken HRCs: 0.19 vs. duck HRCs: 0.18, *p* = 0.012) (Fig. 5c). Additionally, we compared these values to the relative frequencies of poultry core-HRC species, focusing on those shared between both hosts (poultry core-HRCs) and the relative frequencies of core-HRC species uniquely identified in either broiler (chicken unique-HRCs) or ducks (duck unique-HRCs).

Interestingly, a notable contradiction emerged when examining the abundances of rearing-specific core HRC species unique to each bird type. Despite chickens having fewer unique core HRC species than ducks (chicken-specific core HRCs: 8 vs. duck: 14), the relative frequencies of chicken-specific core HRC species were significantly higher than those of duck-specific core HRC species (chicken relative frequency: 0.08 vs. duck: 0.03, p-value = 0.07). This suggests that although ducks have a greater variety of HRC species, their relative abundance is notably lower compared to that of chickens.

### Grower phase microbial biomarkers in broiler- and duck-farm environments exhibiting strong correlations with fluctuations in poultry core HRC species

**Fig. 6:**
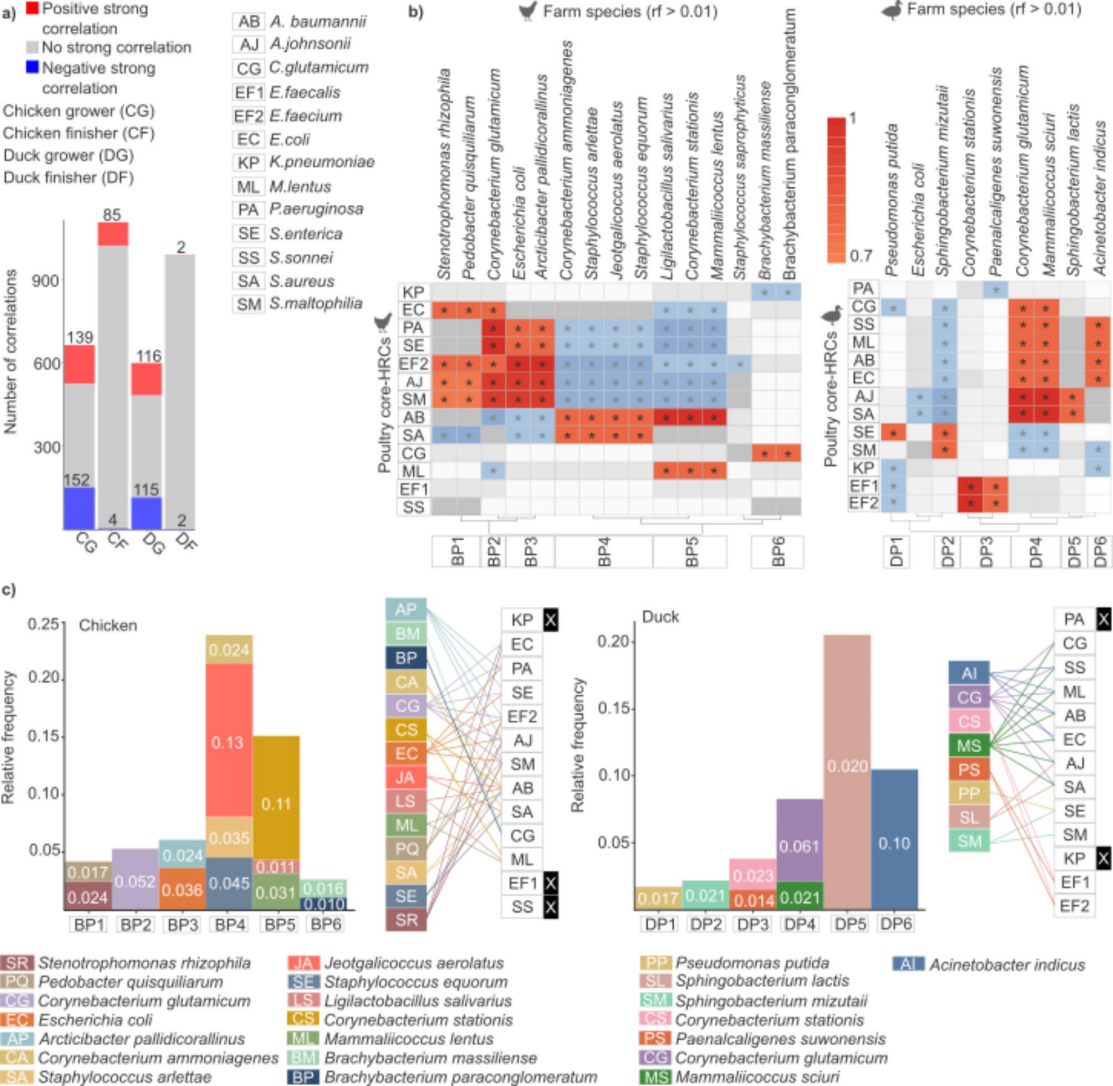
Identification of microbial biomarkers in both broiler and duck environments for tracking poultry core HRC species. (**a**) Bar plots illustrate the number of strong correlations between broiler and duck farm environmental species (relative abundances > 0.001) and the 13 poultry-core HRC species across two production stages (grower and finisher). Positive correlations are indicated in red, negative correlations in blue, and non-significant correlations in gray. (**b**) Clustered heatmaps display the strength of correlations between environmental microbial biomarkers (relative abundances > 0.01) and the 13 poultry-core HRC species in chicken and duck-rearing environments. Positive correlations are represented on a red gradient scale, highlighting significant associations. Fifteen candidate biomarker species were identified in chicken, and nine in duck environments, each showing robust correlations with their respective HRC counterparts. Below the correlation plot, the bars indicate the predefined diagnostic broiler (BP1-BP6) and duck (DP1-DP6) panels. (**c**) Representation of the composition of the six chicken and duck diagnostic panels featuring environmental biomarker organisms exhibiting strong positive correlations with specific HRC species, along with their respective relative frequency values. Black bars with white X represent HRC species that were not detectable with environmental biomarkers. Correlation coefficients were calculated using Spearman’s rank correlation method.

The number of strong correlations between the relative frequencies of farm environmental species with relative abundances exceeding 0.001 and among the 13 poultry-core HRC species was quantified (Fig. 6a). The grower and finisher phases, critical for meat production, were prioritized due to their proximity to the production endpoint and the heightened risk of accumulating production-associated antimicrobial resistance over time^18^. The analysis highlights the grower phase as the most predictive stage for monitoring HRC species. Broiler grower samples exhibited 139 positive correlations (1.64 times more than the finisher phase) and 152 negative correlations (38 times more than the finisher phase). Similarly, duck grower samples showed 116 positive correlations (58 times more than the finisher phase) and 115 negative correlations (57.5 times more than the finisher phase).

Subsequently, by narrowing the focus to potential biomarker species with relative frequencies exceeding 0.01, clustered heatmaps were generated to visualize the correlation patterns between farm environmental microbial biomarkers and the 13 poultry-core HRC species (Fig. 6b). To further enhance the efficiency of monitoring, we only focused on positive correlations to identify the most reliable candidates for tracking any of the poultry-core HRC species through mutual indication. This analysis identified 15 candidate biomarker species in chicken and 9 in duck farm environments, each demonstrating strong correlations (correlation coefficient > 0.7) with their respective HRC counterparts. The paired species names and their corresponding correlation values are detailed in **Supplementary File 4**. The correlation plots revealed rearing-specific farm environmental biomarker organisms with overlapping patterns, indicating potential redundancy. Based on these findings, we organized the biomarkers into six diagnostic panels for broilers (BP1-BP6), and ducks (DP1-DP6).

For each diagnostic panel, we identified the key biomarkers within chicken and duck grower rearing phase that exhibited strong correlations with poultry-core HRC species (Fig. 6c). This targeted approach enabled the identification of the most suitable biomarkers for accurately monitoring HRC species by selecting those with the highest relative frequency values within each panel, ensuring reliable tracking of their HRC counterparts. Our findings indicate that, for each panel, a single biomarker with the highest relative frequency in the broiler grower environment can be prioritized for effective HRC tracking. Accordingly: **Chicken grower environment: Panel 1**: *S. rhizophila* (average rel. freq.: 0.024 ± 0.03) strongly correlates with HRC species *E. coli* (*r* = 0.8), *E. faecium* (*r* = 0.8), *A johnsonii* (*r* = 0.74), and *S. maltophilia* (*r* = 0.74). **Panel 2**: *C. glutamicum* (average rel. freq.: 0.052 ± 0.1) shows a strong correlation with HRC species *E. coli* (*r* = 0.8), *P. aeruginosa* (*r* = 1.0), *S. enterica* (*r* = 1.0), *E. faecium* (*r* = 0.8), *A. johnsonii* (*r* = 0.95), *S. maltophilia* (*r* = 0.95). **Panel 3**: *E. coli* (average rel. freq.: 0.036 ± 0.07) strongly correlates with HRC species *P. aeruginosa* (*r* = 0.8), *S. enterica* (*r* = 0.8), *A.feacium* (*r* = 1.0), *A. johnsonii* (*r* = 0.95), *S. maltophilia* (*r* = 0.95). **Panel 4**: *J. aerolatus* (average rel. freq.: 0.133 ± 0.11) is strongly correlated with HRC species *A. baumanii* (*r* = 0.8) and *S. aureus* (*r* = 0.8). **Panel 5**: *C. stationis* (average rel. freq.: 0.108 ± 0.06) shows a strong correlation with HRC species *(A) baumanii* (*r* = 1) and *M. lentus* (*r* = 0.8). **Panel 6**: *(B) massiliense* (average rel. freq.: 0.016 ± 0.019) strongly correlates with HRC species *(C) glutamicum* ( 0.8). However, relative frequency data from broiler grower phase environmental samples indicate that none of the panels can reliably track changes in HRC species *K. pneumoniae*, *E. Faecalis*, and *S. sonei*, due to insufficient correlation strength.

**Duck grower environment: Panel 1**: *P. putida* (average rel. freq.: 0.017 ± 0.02) shows a strong correlation with HRC species *S. enterica* (*r* = 0.8). **Panel 2**: *S. mizutaii* (average rel. freq.: 0.021 ± 0.02) strongly correlates with HRC species *S. enterica* (*r* = 0.8) and *S. maltophilia* (*r* = 0.8). **Panel 3**: *C. stationis* (average rel. freq.: 0.023 ± 0.004) is strongly correlated with HRC species *E. faecium* (*r* = 1) and *E. faecalis* (*r* = 1). **Panel 4**: *C. glutamicum* (average rel. freq.: ± 0.023) shows a strong correlation with HRC species *C. glutamicum* (*r* = 0.8), *S. sonnei* (*r* = 0.8), *M. lentus* (*r* = 0.8), *A. baumannii* (*r* = 0.8), *E. coli* (*r* = 0.8), *A. johnsonii* (*r* = 1), *S. aureus* (*r* = 1). **Panel 5**: *S. lactis* (average rel. freq.: 0.20 ± 0.17) strongly correlates with HRC species *A. johnsonii* (*r* = 0.8) and *S. aureus* (*r* = 0.8). **Panel 6**: *A. indicus* (average rel. freq.: 0.10 ± 0.08) strongly correlates with HRC species *S. sonnei* (*r* = 0.8), *M. lentus* (*r* = 0.8), *A. baumannii* (*r* = 0.8), *E. coli* (*r* = 0.8). However, in duck grower phase samples, none of the panels can reliably detect changes in the relative frequencies of HRC species *P. aeruginosa* and *K. pneumoniae* due to a lack of strong correlations.

### Distribution of differentially abundant above-average antibiotic resistance markers in broiler chicken and duck biological samples

**Fig. 7:**
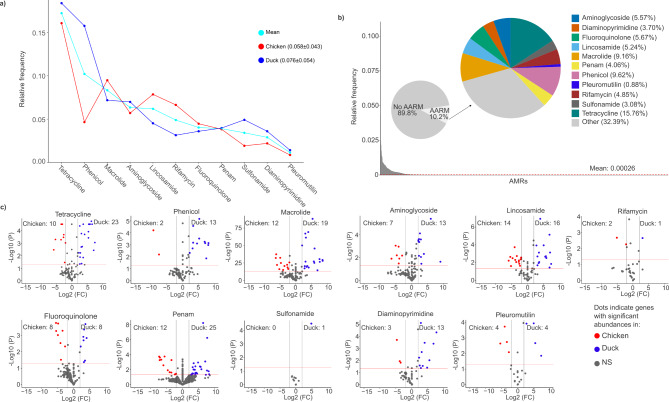
Comparative analysis of antimicrobial resistance classes and above-average resistance markers (AARMs) in chicken and duck. (**a**) The line plot illustrates the relative frequency distribution of the 11 most prevalent antimicrobial resistance classes identified in chicken and duck samples. One line represents the overall mean relative frequency across both species, while the other two depict the class-specific distributions for each species individually. (**b**) Panel (1) Relative frequency of AMRs was visualized on barplot. Above-average antimicrobial resistance markers (AARMs) were defined as those with relative frequencies exceeding the overall average (relative frequency: 0.00026) across all detected AMRs. b, Panel (2) The pie chart displays the proportional distribution of AARMs versus non-AARMs across resistance classes, along with their respective relative frequencies. The bigger pie chart displays the proportion of antibiotic resistance classes in AARMs. (**c**) In the 11 AARMs antibiotic resistance classes volcano plot was utilized to identify AARM genes with significantly different relative abundances between chickens and ducks.

From the current study population, the most abundant resistance classes were identified based on the averaged values in the biological samples of the two bird species, listed in descending order as follows: tetracycline (0.172 ± 0.058), phenicol (0.102 ± 0.078), macrolide (0.083 ± 0.024), aminoglycoside (0.063 ± 0.021), lincosamide (0.062 ± 0.053), rifamycin (0.049 ± 0.038), fluoroquinolone (0.040 ± 0.034), penam (0.039 ± 0.021), sulfonamide (0.034 ± 0.027), diaminopyrimidine (0.029 ± 0.016), and pleuromutilin (0.011 ± 0.010) (Fig. 7a). We also analysed the cumulative AMR values associated with rearing-specific antibiotic resistance classes across the 11 most common classes. Chickens exhibited slightly higher cumulative resistance values compared to ducks (chicken: 0.764, duck: 0.756). In both species, the tetracycline class accounted for the majority of resistances. However, the distribution of other resistance classes showed significant species-specific differences. In ducks, phenicol resistances were nearly as prevalent as tetracycline resistances (tetracycline duck: 0.184 ± 0.054, phenicol duck: 0.158 ± 0.077), standing out as exceptionally high compared to other classes. After a noticeable drop, macrolide and aminoglycoside resistances occurred at nearly equal frequencies (macrolide chicken: 0.094 ± 0.026, macrolide duck: 0.072 ± 0.015; aminoglycoside chicken: 0.057 ± 0.020, aminoglycoside duck 0.063 ± 0.021), followed by sulfonamide resistances, which also had relatively high occurrences (sulfonamide chicken 0.019 ± 0.020, sulfonamide duck 0.049 ± 0.024).In chickens, the trends diverged substantially. While phenicol resistances were the second most common class in ducks, they ranked sixth in chickens, behind macrolide, lincosamide, rifamycin, and fluoroquinolone resistances. Pleuromutilin resistances were the least frequent in both species (pleuromutilin chicken 0.0088 ± 0.0070, pleuromutilin duck 0.014 ± 0.013). These findings highlight distinct resistance class distributions between ducks and chickens, underscoring species-specific dynamics in antimicrobial resistance. An average AMR frequency value was established based on the distribution of all detected AMRs (rf > 0.00026). Following this, above-average antibiotic resistance markers (AARMs) were identified as those exhibiting relative abundance values exceeding this defined average (Fig. 7b).

Our data show that 10.2% of all resistances met the criteria for AARMs, indicating that most (89.8%) of the detected resistances represented below-average relative frequencies. The distribution of AARMs across the most common resistance classes closely aligned with trends seen in the broader resistance dataset. Tetracycline and phenicol AARMs were the most prevalent, with tetracycline representing the largest proportion at 15.76%, followed by phenicol at 9.62%. Diaminopyridine, sulfonamide, and pleuromutilin resistances remained among the least abundant.

Volcano analyses identified AARMs with significant differences in relative abundance between chicken and duck-rearing systems (Fig. 7c). Penam resistance class demonstrated the highest number of differentially abundant AARMs, with 37 markers identified12 more prevalent in chickens and 25 in ducks. Macrolide resistance was followed by 31 differentially abundant AARMs (12 more abundant in chickens and 19 in ducks), while lincosamide resistance included 30 differentially abundant AARMs (14 more prevalent in chickens and 16 in ducks). Aminoglycoside resistance accounted for 20 differentially abundant AARMs, with 7 more abundant in chickens and 13 in ducks. Interestingly, despite high cumulative relative frequencies, rifamycin and sulfonamide resistance classes exhibited the lowest significant differences, highlighting distinct host-specific patterns in resistance class prevalence. These findings underscore the influence of rearing environments on resistance marker distributions across poultry species.

### Host-specific dynamics and species associations of above-average antimicrobial resistances in broiler chicken and duck farming systems

**Fig. 8:**
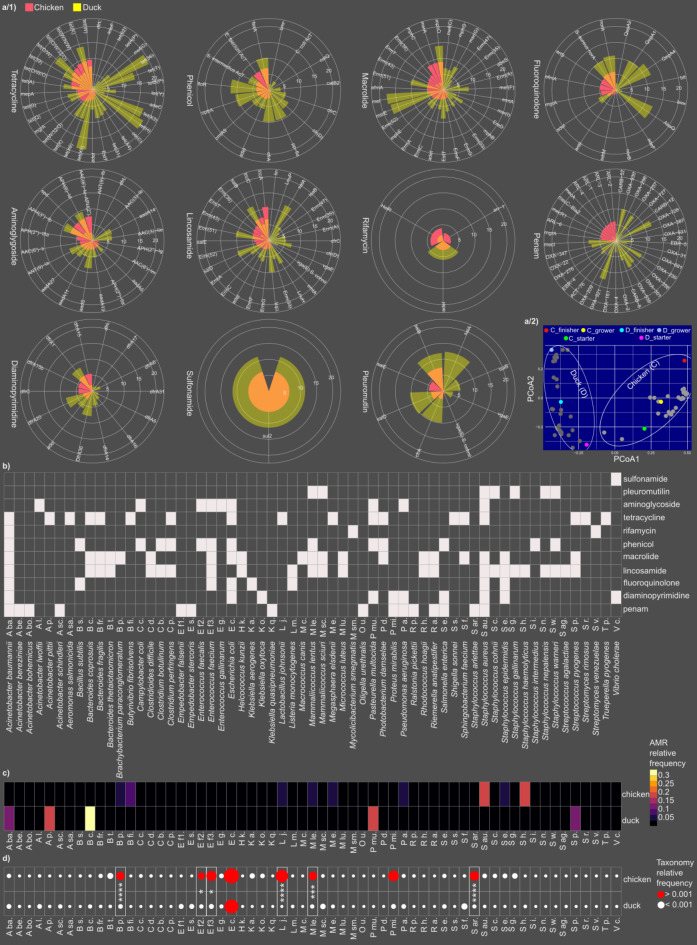
Rearing-specific trends of differentially abundant common antimicrobial resistance markers across resistance classes and host-specific taxonomic insights. (**a**/1) Radar plots illustrate the rearing-specific differences in differentially abundant common antimicrobial resistance markers (AARMs) across various resistance classes. (**a**/2) PCoA plot illustrates the distribution of samples and data pools based on their AARM composition. Grey dots represent chicken and duck manure samples, while colored dots show chicken and duck-rearing data pools. (**b**) Identification of bacteria possibly linked to AARMs. (**c**) Species-level differences in differentially abundant AARM contributions across chicken-, and duck-rearing. d) Relative occurrence of AARM-associated species in broiler and duck manure microbiota. Asterisks indicate significant differences between broiler and duck (* *p* < 0.05, *** *p* < 0.001, ****p < 0.0001).

Detailed analyses of the magnitude of differentially abundant above-average antimicrobial resistances (AARMs) were conducted to quantify the extent of variations between broiler and duck-rearing systems (Fig. 8a**/1**). Based on AARM data analysis, a Principal Coordinates Analysis (PCoA) plot revealed two distinct host-specific clusters, representing the resistomes of broiler chickens and ducks (Fig. 8a**/2**). These clusters demonstrate the unique molecular fingerprints characterising each rearing system. Additionally, the AARM data-based resistome signatures align with production phases, offering valuable insights into the dynamics of antimicrobial resistance throughout the production cycle.

Based on the current study, the analysis of broiler chicken and duck samples identified the significantly differentially abundant resistance genes, based on volcano analysis. The values are visualized as log2 of the read counts. The differences are marked as Δlog2 read count, positive Δlog2 values indicate greater gene abundance in ducks, while negative values signify higher abundance in chicken samples. The Δlog2 values were determined by subtracting the log2 read count the chickens from that in ducks. Tetracycline resistance genes dominated this list, occupying the top five positions. The most abundant gene was *tet36*, encoding a tetracycline-resistant ribosomal protection protein associated with *Bacteroides coprosuis*, which exhibited a Δlog2 read count of 19.46. Following this, *tetH*, linked to *Pasteurella multocida*, encoding a major facilitator superfamily antibiotic efflux pump, had a Δlog2 read count of 17.14. Two flavin-dependent tetracycline inactivation enzyme genes, *tetX3* and *tetX5*, associated with *Acinetobacter baumannii* and *Acinetobacter pittii*, displayed Δlog2 read counts of 15.79 and 15.42, respectively. Lastly, *tetT*, encoding another ribosomal protection protein and linked to *Streptococcus pyogenes*, exhibited a Δlog2 read count of 13.40. Beyond tetracycline resistance, macrolide resistance genes were also prominent. These included *mel*, associated with *Streptococcus pyogenes* (Δlog2 read count: 13.01), and *msrE* (Δlog2 read count: 12.79), linked to *Acinetobacter baumannii*. Both genes encode msr-type ABC-F proteins and were significantly more abundant in chicken samples. Additionally, a fluoroquinolone resistance gene, *abaQ* (Δlog2 read count: 12.64), and a penam resistance gene, *OXA-921* (Δlog2 read count: 12.58), were identified. The final gene in this group was *salA* (Δlog2 read count: 12.32), a lincosamide resistance gene. Among other significantly more abundant genes in chicken samples, several stood out due to their associations with specific bacterial species and resistance mechanisms. Notably, the chloramphenicol acetyltransferase gene, linked to *Enterococcus faecium* and responsible for phenicol resistance, exhibited a Δlog2 read count of 6.62. Similarly, the *msrA* gene, associated with *Staphylococcus epidermidis* and conferring macrolide resistance, had a Δlog2 read count of 6.32. Another macrolide resistance gene, *mphC* (Δlog2 read count: -6.18), connected to *Staphylococcus aureus*, was also identified as significantly more abundant. Following these, four penam resistance genes, *ARL1-4* beta-lactamase (mean Δlog2 read count: -5.81), associated with *Staphylococcus arlettae*, were among the most differentially abundant resistance genes in chicken samples.

Through in silico analyses, we further explored potential associations between AARMs and their corresponding resistance-carrying species (Fig. 8b). *Acinetobacter baumannii* and *Staphylococcus aureus* were linked to the highest number of differentially abundant AARMs, each associated with eight distinct resistance classes. Among these, the penam, diaminopyridine, fluoroquinolone, lincosamide, macrolide, and tetracycline resistance classes co-appear in both pathogen species. Additionally, *Escherichia coli* emerged as a potential carrier of six resistance classes, followed by *Enterococcus faecium*, which was associated with five. Meanwhile, *Staphylococcus epidermidis*, *Mammaliicoccus lentus*, *Pasteurella multocida*, and *Staphylococcus aureus* were each linked to four distinct resistance classes.

We identified the bacterial species most strongly associated with the highest number of AARMs in both chicken and duck (Fig. 8c). In ducks, *Bacteroides coprosuis*, *Acinetobacter pittii*, *Pasteurella multocida*, *Acinetobacter baumannii*, and *Streptococcus pyogenes* were associated with the highest number of AARMs (*Bacteroides coprosuis*: 0.33, *Acinetobacter pitii*: 0.19, *Pseudomonas mirabilis*: 0.18, *Acinetobacter baumanii*: 0.13, *Streptococcus pyrogenes*: 0.11). In chickens, by contrast, AARM frequencies were generally lower, with *Staphylococcus aureus*, *Staphylococcus haemolyticus*, *Butyrivibrio fibrisolvens*, *Lactobacillus johnsonii*, *Mammaliicoccus lentus*, and *Megasphaera elsdenii* showing the highest AARM values (*Staphylococcus aureus*: 0.19, *Staphylococcus haemolyticus*: 0.17, *Butyrivibrio fibrisolvens*: 0.082, *Staphylococcus epidermidis*: 0.059, *Mammaliicoccus lentus*: 0.056, *Megasphaera elsdenii*: 0.041).

Subsequently, taxonomic data of differentially abundant AARM-associated species in broiler and duck manure microbiota were also compared (Fig. 8d). It is noteworthy that in ducks, the species associated with the highest AARM loads exhibited relatively low occurrence rates compared to other potential AARM carrier organisms (average relative frequencies of highlighted species: 0.093 vs. the overall average relative frequencies of all AARM-carrying species: 0.016) suggesting these species, despite their lower prevalence, playing a disproportionately significant role in contributing to the AARM burden in ducks. Notably, despite *E. coli* exhibiting a relatively high relative frequency in ducks, its association with AARMs was negligible. A similar trend was observed in chickens, where *E. coli* had the highest frequency among potential AARM carriers but demonstrated minimal associated AARM abundances.

Based on current study data, seven AARM carrier species whose relative abundance was significantly higher in broiler chickens compared to ducks were identified. These species included *Brachybacterium paraconglomeratum* (*p* < 0.0001), *Enterococcus faecalis* (*p* = 0.001), *Enterococcus faecium* (*p* = 0.016), *Lactobacillus johnsonii* (*p* < 0.0001), *Macrococcus lentus* (*p* = 0.0003), and *Staphylococcus arlettae* (*p* < 0.0001). These findings underscore host-specific differences in antimicrobial resistance dynamics between broiler chickens and ducks rearing systems.

## Discussion

Antimicrobial resistance is a pressing global challenge that demands a comprehensive and coordinated response across public health sectors, encompassing human, animal, and environmental domains. The poultry industry, a cornerstone of global food security, faces significant challenges due to the widespread use of antibiotics, particularly for prophylactic purposes, disease control, and as growth promoters^19,20^. These practices contribute to the selection and dissemination of multidrug-resistant bacteria, which can have profound implications for public health and the sustainability of poultry farming^19,20^.

AMR in poultry farming is not an isolated issue; it directly threatens human health. Resistant bacteria from poultry can transfer to humans through multiple pathways, including the food chain, direct animal contact, and environmental exposure^20^. The presence of such bacteria in food products heightens the risk of treatment failures in human medicine, complicating the management of infections and increasing morbidity, mortality, and healthcare costs^20^. The intersection of human and animal health highlights the urgent need for integrated approaches under the One Health framework.

The poultry industry plays a pivotal role in global protein production being central to feeding a growing population^5^. However, its dependency on antibiotics to ensure high productivity and prevent disease outbreaks presents a dual challenge: maintaining animal health and performance while mitigating the risks of AMR^5^. Failure to address AMR effectively could lead to stricter regulations, higher production costs, and decreased consumer confidence, all of which threaten the industry’s long-term viability.

Robust data collection on antibiotic usage and resistance patterns is critical to understanding the drivers of AMR in poultry production. Such information can inform evidence-based policies, encourage the adoption of best practices, and guide interventions aimed at reducing antibiotic reliance. By fostering transparency and accountability, these efforts can help strike a balance between safeguarding animal health and minimizing the public health risks associated with AMR.

Hungary’s veterinary antibiotic usage, with an mg/PCU value of 69.9 (ESVAC, 2010–2022), ranks mid-level among 31 European countries. While Nordic countries such as Norway and Denmark report significantly lower values, higher usage is observed in countries like Italy (157.5), Spain (127.3), and Poland (123.5). Hungary’s total sales of 120 tonnes are moderate compared to Spain’s 1,027.2 tonnes and Italy’s 585.4 tonnes^21^.

However, challenges remain, particularly in addressing historical antibiotic practices, regional variability in compliance, and environmental reservoirs of resistance. As Hungary navigates the global AMR crisis, it underscores the need for integrated, cross-sectoral approaches that prioritize sustainability and public health.

Research on antimicrobial resistance in poultry predominantly focuses on chickens, with significantly less attention given to ducks. Approximately 85–90% of poultry-related AMR studies concentrate on chickens, while only 10–15% or fewer investigate ducks. This disparity reflects the global economic and dietary importance of chickens, positioning them as the primary model for AMR research^7,22^.

Therefore, the primary aim of our study was to conduct a comprehensive investigation of antimicrobial resistance dynamics in broiler and duck production systems across broiler chicken and duck production stages. Samples were collected from intensively reared poultry species—Ross 308 broilers and Cherry Valley ducks—from Hungerit Zrt’s farms in Szentes and Kistelek, Hungary. Consistent rearing parameters, including standardized housing technologies and management practices, were maintained throughout the 15-month study period, minimizing potential distortion factors arising from variations in rearing technologies. The extended duration, encompassing 15 stocking periods, provided a representative dataset, enabling meaningful comparisons between the two poultry production systems.

In this longitudinal study, despite temporal variations, key factors such as hatchery practices, rearing conditions, feed formulations, and breeds were maintained consistently, complying with EU standards to ensure the reliability of our findings and their relevance to the investigated poultry production systems.

With this, we aimed to reflect the realities of intensive poultry farming in Hungary and the EU. There were 3665 antimicrobial resistance types identified across broiler and duck-rearing, highlighting significant differences in their AMR profiles. Ducks exhibited substantially higher overall AMR levels, while broilers demonstrated a broader spectrum of resistance. This indicates that in ducks, resistance was concentrated within a smaller subset of AMR carrier microbes, whereas in broilers, resistance was more evenly distributed across a larger pool of carrier species.

The observed differences in AMR trends between chickens and ducks can be attributed to several factors, including distinct host-associated microbiomes, environmental conditions, and feed compositions. Host microbiomes play a central role in shaping species-specific resistomes, with ducks and broilers exhibiting unique microbial communities that influence the accumulation and transmission of resistance determinants, resulting in characteristic AMR profiles for each species.

Early exposure to environmental resistance markers, such as those encountered during transportation from hatcheries, often shapes resistomes^23^. However, in our case, this factor was not relevant, as Hungerit Zrt., the company responsible for broiler chicken and duck meat production, operates its own breeding stock and hatchery.

Despite biosecurity measures, airborne AMR genes can integrate into commensal or pathogenic bacteria, establishing an initial resistance burden^24^. Upon arrival on the farm, resistomes are further influenced by farm-specific conditions, management practices, and prior antimicrobial use.

Ducks exhibited significantly higher overall AMR levels, while broilers displayed a broader spectrum of resistance. In ducks, resistance was concentrated in a smaller subset of AMR carrier microbes, whereas in broilers, resistance was distributed across a wider range of species. Environmental factors also play a critical role; ducks are reared in wetter conditions that promote bacterial proliferation, biofilm formation, and the spread of resistance determinants^17^.

Feed composition further contributes to resistance dynamics, particularly through the presence of heavy metals like zinc and copper, which exert selective pressure^25^. Duck feed contained 1.03 times more zinc and 1.2 times more copper than broiler feed, promoting resistance accumulation via co-selection and cross-resistance mechanisms. Across production stages, broiler starter feeds contained the highest concentrations of these metals, with levels 1.1–1.4 times higher than grower feeds and twice those of finisher feeds. In ducks, the elevated zinc and copper content in the two-phase starter feed similarly drove resistance increases during the grower stage. However, heavy metal reductions in duck feed began earlier in the grower phase, leading to a noticeable decrease in AMR levels by the finisher stage, mitigating some of the microbiome-altering effects. Host-specific core and unique resistances were also investigated. Common core AMRs accounted for about 57% of all detected types, while broilers exhibited a higher proportion of host-specific unique AMRs compared to ducks. This suggests that in chickens, the development of newly acquired resistances might occur to a greater extent, likely driven in part by rearing-specific selection pressures. These resistances are typically present at low frequencies, as further supported by the observation that the Shannon diversity of species potentially linked to AMRs was significantly lower in chickens than in ducks. This difference can be attributed to the fact that the Shannon index considers both richness and evenness, and the significantly lower values observed in chickens are likely due to the much less balanced distribution of taxonomic data.

Despite the higher overall resistance in ducks, broilers exhibited a greater proportion of host-specific AMRs. Notably, AMR diversity declined significantly from the starter to the finisher phase in both species, with broilers experiencing a more pronounced reduction, potentially due to the presence of increasingly less dynamic AMR carrier microbial communities. Conversely, AMR relative frequencies increased during the finisher phase in both species, reflecting an inverse relationship between diversity and abundance. This trend can be attributed to reduced competition among microbial taxa; as less dominant populations diminish, resistant strains proliferate, leading to their dominance in the microbial community. Prophylactic antibiotic use in Hungary is strictly regulated, limited to justified cases, and applied intermittently in compliance with EU guidelines^26^. During our study both broiler and duck rearing systems included proportional numbers of flocks subjected to prophylactic antibiotic treatments: 3 out of 8 production cycles in broiler chickens and 3 out of 7 in ducks. Ducks received sulfonamides, diaminopyrimidines and fluoroquinolones, while broilers were treated with fluoroquinolones, phencicols, sulfonamides, and diaminopyrimidines. Notably, phenicol use is of particular interest, as this resistance class emerged as the second most prevalent overall in ducks, whereas in broilers, phenicol resistance ranked only fifth, following macrolide, lincosamide, rifamycin, and aminoglycoside resistances.

While no distinct clustering was observed in the resistance patterns associated with multidrug resistant bacteria between antibiotic-treated and antibiotic-free flocks, a significant difference emerged in the prevalence of MDRBs. In the affected stocking periods, antibiotic treatments accounted for an average of 11.90% ± 2.38% of the stocking period in broiler chickens and 10.32% ± 2.75% in ducks. Based on this, in both systems, flocks receiving prophylactic antibiotic treatments for approximately 2–3% of the rearing phase showed significantly lower occurrences of MDRBs compared to untreated flocks, highlighting the beneficial role of rational antibiotic use.

When applied judiciously, prophylactic antibiotics can effectively suppress opportunistic pathogens, fostering a more balanced and stable microbial environment within the host^27^. The reduction in pathogenic bacterial load can directly influence the dynamics of resistance accumulation by diminishing the microbial reservoirs that harbor resistance genes^28^. Furthermore, since antibiotic treatments target both resistant and non-resistant bacterial populations, they can effectively lower overall bacterial density which reduction can decrease the probability of horizontal gene transfer^29^.

The interplay between the host microbiome and its environment further underscores the observed reductions. In antibiotic-treated flocks, similar to the samples, the immediate environment (e.g., bedding, water) likely harbors fewer resistant bacteria, reducing opportunities for re-colonization by multidrug-resistant organisms and mitigating the risk of external amplification of resistance determinants.

Hospital-acquired infection-associated bacterial species represent a significant proportion in our analysis, particularly in chicken samples underscoring the role of post-hatch poultry, especially broilers, as potential reservoirs for HAI-associated AMR genes. These patterns can likely be associated to either inadequate sanitation in hatcheries or suboptimal conditions during transportation or both^23^. Poor ventilation and prolonged transit times can exacerbate bacterial transmission and increase stress-induced susceptibility to resistant strains^23^. The proportion of HAI-associated AMRs was markedly higher in broiler chickens than in ducks at the beginning of the production. However, a reassuring trend was observed in both rearing systems, as these proportions declined significantly by the end of the rearing periods. This reduction may be attributed to proper hygiene practices and strict adherence to prescribed standards implemented during the production process^30,31^.

In contrast, the proportions of AMRs associated with foodborne pathogens were relatively negligible. Our analysis of AMR distribution among commensals and foodborne pathogens revealed no significant differences across rearing phases. Although the prevalence of AMRs linked to commensal bacteria was relatively low in both poultry species, broilers displayed greater diversity in the AMR types associated with these beneficial microorganisms. Short-chain fatty acid producing bacteria, which are essential for gut health and host immunity, also showed associations with AMRs. Furthermore, the elevated production of SCFAs, particularly during the finisher phase, may indicate the presence of inflammation, a common occurrence in broilers during the grower and finisher phases^32^. This observation aligns with findings from our previous study, which highlighted inflammation-associated shifts in gut microbiota during intensive broiler production^33^.

One of the central focus of our study was the identification of high-resistance carrier species within both rearing systems and their environmental sensory counterparts. We highlighted the role of HRC species in shaping resistance profiles. While broilers exhibited a larger HRC pool (43 species) compared to ducks (36 species), the cumulative AMR abundance within these pools was higher in ducks. Broilers exhibited 21 distinct species, whereas ducks demonstrated 27 species meeting the same criteria. Notably, despite the lower number of rearing-specific HRC species identified in broiler production, their cumulative abundance values were significantly higher than those observed in duck production. This finding suggests that in broilers, the majority of AMRs are concentrated in a small number of dominant microbial species, while the remaining resistances are associated with low-abundance, less prevalent taxa. In contrast, in ducks, the majority of AMRs are predominantly linked to microbial species shared with broilers. Duck-specific AMRs, defined by resistance profiles unique to ducks, contribute minimally to the overall resistance burden, underscoring their limited impact on the broader AMR landscape within duck production systems.

Thirteen poultry core HRC species were consistently detected across broiler and duck production systems in all rearing phases, collectively harboring the majority of AMR determinants and highlighting their role in AMR management. While ducks had a larger HRC species pool, broilers displayed higher cumulative relative abundances among their unique HRC species. To enhance AMR monitoring, we identified environmental biomarkers that strongly correlate with shifts in HRC prevalence. These biomarkers could serve as effective proxies for tracking AMR dynamics. A set of 15 biomarker species in chicken environments and 9 in duck environments demonstrated strong correlations (correlation coefficient > 0.7) with the poultry-core HRCs.

Among the three rearing sub-stages—starter, grower, and finisher—the grower phase emerged as the most suitable for monitoring due to its transitional nature, where targeted interventions can still be effectively implemented to mitigate the spread of antimicrobial resistance.

Environmental biomarkers were highly effective in tracking poultry-specific HRC species during the grower phase for both chickens and ducks. However, limitations were observed: in chickens, *Klebsiella pneumoniae*, *Shigella sonnei*, and *Enterococcus faecalis* were not reliably trackable, while in ducks, only *Klebsiella pneumoniae* and *Pseudomonas aeruginosa* could not be monitored. These findings underscore the utility of environmental biomarkers in AMR surveillance and highlight the grower phase as a critical window for intervention.

We also focused on above-average resistances, which accounted for approximately 10% of all detected resistance determinants. These AMRs were considered representative, as their distribution across the top 11 resistance classes mirrored the overall AMR population, excluding transient low-frequency resistances. Among the most prevalent resistance classes were tetracycline, phenicol, macrolide, disinfecting agents, and fluoroquinolone, reflecting their widespread use in poultry production. In contrast, diaminopyrimidine, sulfonamide, and pleuromutilin resistances were rare. Our comparative analyses revealed dynamic shifts in resistome profiles across production phases and distinct host-specific molecular fingerprints in broiler chicken and duck systems. Tetracycline and phenicol resistances emerged as the most abundant AARMs, dominating both systems. Penam resistance exhibited the greatest variation, with markers differentially distributed between the two hosts. While rifamycin and sulfonamide classes showed minimal significant differences, tetracycline resistance markers were consistently more abundant in broiler systems, highlighting host-specific selective pressures. Species-level analyses further emphasized these host-specific resistome characteristics. In broilers, species such as *Staphylococcus arlettae*, *Lactobacillus johnsonii*, and *Enterococcus faecium* were major contributors to AARM profiles, showcasing the concentrated AMR burden in these taxa. Conversely, duck-specific AARMs were linked to less prevalent species, such as *Bacteroides coprosuis* and *Acinetobacter pittii*, which disproportionately contributed to the duck resistome. *Acinetobacter baumannii* and *Staphylococcus aureus* were notable in both systems, each associated with eight resistance classes, underscoring their critical role in AMR dissemination. Interestingly, *Escherichia coli*, despite its high relative abundance, showed negligible contributions to AARMs.

A comprehensive U.S. study on chicken and turkey production systems provides valuable pathogen-specific AMR insights based on a large-scale temporal dataset spanning nearly a decade (2015–2023)^34^. This study identified *Salmonella enterica*, *Campylobacter jejuni*, and *E. coli/Shigella* as the primary AMR pathogens. Similarly, our research on broiler and duck production systems identified *S. enterica*, *E. coli*, and *Shigella sonnei* as significant pathogens. However, *Campylobacter jejuni* was notably absent as a high-resistance carrier species in our systems. The U.S. study highlighted tetracycline and streptomycin resistance as the most prevalent types, alongside geographic hotspots for resistance genes^34^. In contrast, while tetracycline resistance was also dominant in our study, phenicol and macrolide resistance were equally prominent. Furthermore, the U.S. research documented resistance genes like *tet(A)* and *mdsB* in both chickens and turkeys, with *blaTEM* being more common in turkeys^34^. In comparison, our study revealed significant host-specific differences in tetracycline resistance genes, such as *tet(36)*,* tet(H)*,* tet(X5)*,* tet(X3)*,* tet(O/W)*,* tet(K)*, and *tet(T)*, in addition to *tet(A)*. Variations in macrolide resistance were also observed, with *meI* and *msrE* differing significantly between broiler and duck systems.

## Conclusion

Antimicrobial resistance is a critical global health challenge that demands coordinated efforts across multiple sectors. Poultry production systems are significant contributors to AMR dynamics, highlighting the need for targeted interventions. Hungary serves as a valuable case study within the European context, with its moderate veterinary antibiotic usage (69.9 mg/PCU) reflecting efforts to align with European Food Safety Authority (EFSA) guidelines. By focusing on both broiler chickens and ducks—two economically important but differently studied poultry species—this research addresses a notable gap in AMR studies, which have traditionally centered on chickens. The here introduced resistome analyses enabled a detailed comparison of resistance distributions between the two rearing systems, revealing key differences influenced by farming practices. Ducks exhibited higher overall resistance abundance, while broilers demonstrated a broader spectrum of unique resistances. Notably, judiciously applied prophylactic antibiotic treatments were shown to significantly reduce the abundance of multidrug-resistant bacteria, underscoring the importance of rational, evidence-based antibiotic management practices. The study’s longitudinal design, incorporating full-cycle daily monitoring and critical pre-slaughter sampling, provided a robust dataset capturing the temporal dynamics of resistance patterns. The grower phase emerged as a pivotal window for AMR mitigation, with environmental biomarkers identified as valuable tools for monitoring resistance spread. This study provides insights into AMR dynamics in poultry production. It examines both zoonotic and commensal bacteria (e.g., *E. coli*,* Salmonella*,* Campylobacter*, and *Enterococcus*), identifying trends and potential links between animal and human health.

While the study spans 15 months and 15 production cycles, its representativeness could be further enhanced with a larger sample size and extended monitoring period. Although significant differences between broiler and duck resistomes were identified, underlying host-specific mechanisms, such as microbial interactions and immune responses, remain underexplored. For multidrug-resistant bacteria, the current findings are based on in silico analyses; incorporating complementary culturomics approaches, where individual microbial community members are cultured and their resistance phenotypes tested, would provide additional depth. Finally, while our study reflects real-world scenarios on commercial broiler chicken and duck production, the absence of controlled experimental setups limits the ability to isolate specific factors influencing AMR dynamics. Despite its limitations, this research offers valuable insights into the comparison of AMR trends between two commercially significant poultry types, paving the way for more targeted interventions and sustainable antimicrobial management strategies in poultry production.

## Materials and methods

### Birds and housing

In our study biological (animal droppings) and environmental samples (farmhouse indoor surfaces) from intensively reared poultry species, namely Ross 308 broilers and Cherry Valley ducks were collected between 2022 and 2024 from Hungerit Zrt’s farms in Rém, Kistelek and Felgyő (Gps coordinates: 46.65513817352346, 20.271366227943012).

In both the broiler chicken and duck farms, 10 sheds were used for rearing the birds. Sampling for biological replicates was conducted in parallel during production. Accordingly, sample pools were collected from sheds no. 9 and 10 for broiler chickens, and sheds no. 4 and 5 for ducks. Both broilers and ducks were housed in two parallel sheds, each being a large facility capable of accommodating approximately 30,000 chickens or 8,000 ducks, depending on the species. The animals were reared under standard management conditions in thermostatically controlled environments. The broilers were kept in floor pens covered with wood shavings, while the ducks were housed in total-confinement duck facilities. To enhance the representativeness of the findings, samples were collected from parallel sheds, facilitating the comparability of commercial intensive poultry production systems and enabling meaningful comparisons. The company also operates its own breeding stock and hatchery. This ensures that day-old poultry (both ducks and chickens) are bred in-house following established protocols and delivered to farms under strictly controlled conditions.

For broilers, the average flock size per stocking period was 28,836 birds. Stocking densities varied across rearing phases: starter phase 1 (3.55 kg/m^2^ in shed no. 9), starter phase 2 (9.29 kg/m^2^ in shed no. 10), grower phase 1 (18.08 kg/m^2^ in shed no. 9), grower phase 2 (27.68 kg/m^2^ in shed no. 10), finisher phase 1 (36.68 kg/m^2^ in shed no. 9), and finisher phase 2 (36.99 kg/m^2^ in shed no. 10).

For ducks, the average flock size per stocking period was 7,137 birds. Stocking densities also varied by rearing phase: starter phase 1 (1.74 kg/m^2^ in shed no. 4), starter phase 2 (4.89 kg/m^2^ in shed no. 5), grower phase 1 (9.29 kg/m^2^ in shed no. 4), grower phase 2 (13.68 kg/m^2^ in shed no. 5), finisher phase 1 (18.25 kg/m^2^ in shed no. 4), and finisher phase 2 (19.63 kg/m^2^ in shed no. 5).

The study was approved by the institutional ethics committee of the University of Debrecen (ethical permission number: 5/2021/DEMÁB). All methods were performed following the relevant guidelines and regulations.

### Diet composition and feeding practices

Birds were fed species-specific diets formulated in-house by the production company to meet the nutritional requirements for each feeding phase: Duck-pre-starter, Duck-starter, Duck-grower I, and Duck-grower II for ducks, and Chick-starter, Chick-grower I, Chick-grower II, and Chick-finisher in case of chickens. The nutritional composition of the feed is provided in **Supplementary File 5** for detailed reference. Feed and water were provided ad libitum.

### Antibiotic treatment

The use of antibiotics in livestock (ducks, chickens) varied during the study. Antibiotic treatments were carried out per EU regulations and veterinary treatment instructions, ensuring in all cases the maximum doses recommended for optimal therapeutic efficacy the and the maximum doses recommended for optimal therapeutic efficacy in all cases. The treatments were also carried out in parallel sheds in every instance.

### Chicken

During the livestock stocking, antibiotic treatments were applied to chicken in the 1st, 2nd, and 8th stocking periods.

During the first stocking period (starter phase 2), an average antibiotic dosage of 10.99 mg per animal per day (Sulfacloroquine-Na, Trimethoprim) was administered for three days. The average body weight ranged from 0.18 to 0.5 kg, and the average number of avians was 25,392. In addition, during the second treatment (grower phase 2), an average antibiotic dosage of 10.01 mg per animal per day (Enrofloxacin) was administered for three days. The average body weight ranged from 0.9 to 1.2 kg, and the average number of avians was 24,986.

In the second stocking period (grower phase 1), an average antibiotic dosage of 5.52 mg per animal per day (Enrofloxacin) was administered for four days. The average body weight ranged from 0.46 to 0.64 kg, and the average number of avians was 25,382.

In the eighth stocking period (starter phase 1 and 2), an average antibiotic dosage of 1.65 mg per animal per day (Enrofloxacin) was administered for five days. The average body weight ranged from 0.1 to 0.21 kg, and the average number of avians was 31,500.

In the affected stocking periods, the duration of antibiotic treatments accounted for an average of 11.90% ± 2.38% of a stocking period in chickens.

### Duck

Antibiotic treatments were applied to ducks in the 2nd, 5th and 7th stocking periods during the livestock stocking.

During the second stocking period (grower phase 1), an average antibiotic dosage of 14.47 mg per animal per day (Florfenikol) was administered for five days. The average body weight ranged from 0.7 to 1.5 kg, and the average number of avians was 5,668.

In the fifth stocking period (grower phase 1), an average antibiotic dosage of 17.44 mg per animal per day (Florfenikol) was administered for five days. The average body weight ranged from 0.74 to 1.42 kg, and the average number of avians was 6,888.

In the seventh stocking period (grower phase 2), an average antibiotic dosage of 48.17 mg per animal per day (Sulfacloroquine-Na, Trimethoprim) was administered for three days. The average body weight ranged from 1.4 to 2.1 kg, and the average number of avians was 7,473.

In the affected stocking periods, the duration of antibiotic treatments accounted for an average of 10.32% ± 2.75% of a stocking period in ducks.

### Sample collection

Biological-footbag (manure) and environmental-footbag (site corridor) samples were collected at the age of 1, 2, 3, 4, 5 and 6 weeks (starter 1, starter 2, grower 1, grower 2 and finisher 1, finisher 2 sampling periods). Chickens had a total of 8 stocking periods, while ducks had a total of 7 stocking periods examined.

Continuous monitoring was carried out at one stocking period per species (duck 2nd, chicken 4th stocking period), in parallel sheds, covering all sampling periods (starter 1, starter 2, grower 1, grower 2 and finisher 1, finisher 2). During the others stocking periods (duck 1st, 3rd, 4th, 5th, 6th and 7th, chicken 1st, 2nd, 3rd, 5th, 6th, 7th, 8th stocking period), we only examined the last finisher periods (finisher 2 sampling periods).

Due to the hygienic criteria of the livestock farm and poultry barn, and the maintenance of a sterile environment (pathogens outside the farm), manure samples were collected with textile footbags that fit the footwear. During sampling, poultry barns were carefully walked around to ensure homogeneous sampling. In parallel with the manure samples, environmental footbag samples were also collected from the site corridor with the same method and procedure, in areas where only the farm workers had access. Both biological and environmental samples were stored in individually marked lockable sterile bags and transported on ice to the laboratory of the University of Debrecen, Faculty of Agricultural and Food Sciences and Environmental Management, Center for Complex Systems and Microbiome Innovations. The samples were stored at -80 °C until pooling and DNA extraction. The biological and environmental samples were pooled by week, with each pool consisting of 7 subsamples.

During the experiment 96 samples were collected, a total of 50 pooled samples from chicken barns (26 biological and 24 environmental pooled samples). A total of 46 pooled samples from duck barns (24 biological and 22 environmental pooled samples). The discrepancy in the number of pooled manure samples compared to farmhouse samples for both poultry species is attributed to unsuccessful analyses caused by suboptimal sequencing results. In duck houses, the excluded samples corresponded to farmhouse samples from the finisher 2 phase (week 6) of the second stocking period. In chicken houses, the missing samples were from the starter 2 phase (week 2) of the fourth stocking period.

### Sample preparation and mechanical cell lysis

Bacterial cell suspensions were made from environmental and biological-footbag samples (one pooled sample consists of 7 subsamples) and homogenized with 40–40 ml of sterile PBS (biosera, Cholet, France) for 30 min at 240 x g, at 30 °C by innova 40 (Incubator Shaker Series). Supernatants were centrifuged for 10 min at 10,000 × g (Centrifuge 5810 R, Eppendorf, Hamburg, Germany). Finally, the supernatants were discarded, and the bacterial pellets were then resuspended in 3 ml of sterile PBS buffer. One-milliliter aliquots of bacterial cell suspensions were transferred to PowerBead tubes (Qiagen, Hilden, Germany) for mechanical cell lysis. Before the mechanical cell lyses the samples were centrifuged at 16,000 x g for 5 min (Centrifuge 5418, Eppendorf, Hamburg, Germany), and the supernatants were discarded. The process was repeated twice, utilizing the entire volume of the bacterial cell suspension. After 1000 µL of InhibitEX buffer (Qiagen GmbH, Hilden, Germany, Lot No. 175011763) were added to the bacterial cell suspensions pellet. Bacterial cell disruption was carried out using a MagNA Lyser instrument (Roche Applied Sciences, Penzberg, Germany) at 4,000 x g for 30 s. The samples were incubated at 4 °C for 2 min and repeated the MagNA Lyser instrument process. The samples were incubated at 95 °C and 800 x g for 7 min by Eppendorf ThermoMixer^®^ C (Eppendorf, Hamburg, Germany). Then centrifuged for 1 min at 15,000 × g at room temperature to pellet the DNA and 400 µL supernatant was transferred to new microcentrifuge tubes (Qiagen GmbH, Hilden, Germany, Lot No. 075826) carefully without disturbing the pellet.

### DNA extraction

DNA extraction from bacterial cell suspension samples was performed via the QIAamp Fast DNA Stool Mini Kit (Qiagen, Germany, Cat. 51604) following the manufacturer’s instructions. Minor modifications were made to optimize the DNA extraction. DNA concentrations were determined fluorometrically via a Qubit^®^ Fluorometric Quantitation HS dsDNA Assay (Invitrogen by Thermo Fisher Scientific, Cat. 2600187) kit with a Qubit^®^ 4.0 fluorometer (Thermo Fisher Scientific, USA).

During the process, we added 30 µL of Proteinase K solution (Lot No. 175028761) and 400 µL of Buffer AL Lysis (Lot No. 172045756) buffer to the 400 µL of supernatant (microcentrifuge tube), and homogenized it for 5 s using vortexing (FV-2400 Minicentrifuge-Vortex Microspin, Biosan, Latvia, Riga). The samples were incubated at 70 °C 10 min by Dry Block Thermostat (Bio TDB-100, Biosan, Latvia, Riga). After incubation, we added 400 µL of 96–100% sterile ethanol to the microcentrifuge tubes. 650 µl of lysate from a microcentrfuge tube was carefully applied to the QIAamp Mini Spin Column (Lot No. 175028898). The cap was closed, and the samples were centrifuged at 15,000 × g at room temperature for 1 min (Centrifuge 5418, Eppendorf, Hamburg, Germany). Then the filtrate was removed from the collection tube. The remaining lysate from the microcentrifuge tube was applied to the Mini Spin Column, the cap was closed, and it was centrifuged at 15,000 × g at room temperature for 1 min. The Mini Spin Column was then placed in a new 2 ml Collection Tubes (Lot No. 175027084), and the tube containing the filtrate was discarded. Then 500 µl of Buffer AW1 (Lot No. 172047716) was added to the Mini Spin Column. It was centrifuged at 15,000 × g at room temperature for 1 min and the filtrate was removed from the Collection Tube. Then 500 µl of Buffer AW2 (Lot No.172042134) was added to the Mini Spin Column. It was centrifuged at 15,000 × g at room temperature for 1 min, the Mini Spin Column was then placed in a new 2 ml Collection Tubes, and the tube containing the filtrate was discarded. During the drying step, the samples were centrifuged at 15,000 × g at room temperature for 3 min, the tube containing the filtrate was discarded and Mini Spin Column was then placed in a new 1,5 ml Micro Tube (Lot No. 0083521). Then 50 µl of Buffer AE (Lot No. 157043769) was added to the Mini Spin Column. The samples were incubated at room temperature for 3 min and centrifuged at 15,000 × g at room temperature for 1 min. The Mini Spin Columns were discarded, and the eluted DNA samples (1.5 ml Micro Tube) were stored at − 20 °C.

### Sequencing and metagenomic data processing

Shotgun sequencing was conducted on an Illumina NovaSeq 6000 instrument (Illumina, USA) with a 150-bp paired-end sequencing run at Novogene Bioinformatics Technology (Beijing, China). The sequencing yielded a minimum of 20 million reads per sample. To ensure the availability of 20 million reads per sample, we reisolated each sample until the required purity (OD260/280 = 1.8–2.0) and concentration ≥ 10 ng/µL for each metagenomic isolate were obtained. Microbial bioinformatics analysis was completed via the SqueezeMeta pipeline (v1.6.3), which uses the coassembly option with no binning^35,36^. The raw reads were quality-filtered with Trimmomatic software (v.0.39) with the following settings: LEADING:8 TRAILING:8 SLIDINGWINDOW:10:15 MINLEN:30. Briefly, paired-end reads were assembled via Megahit. For taxonomic assignment DIAMOND v.2.19 runs were performed against GenBank nr database using a fast LCA algorithm^37–40^. We used the KIFÜ Hungarian High Performance Computing Competence Center (HPC CC) Komondor HPC with 48 CPU cores and 90 GB of RAM per sample. For the bioinformatics analysis of antibiotic resistance, KneadData software was used for quality control of the sequencing data, utilizing Trimmomatic and Bowtie2^41,42^. RGI software, in conjunction with the CARD database, was used to predict the antibiotic resistome^43,44^.

### Statistical analysis and data visualization

Continuous variables were reported as the mean ± standard deviation. The Wilcoxon rank-sum test was applied to statistically compare continuous variables. Continuous variables, for example, the relative frequency of AMR, were always highlighted. All statistical tests were two-tailed, with a significance level set at *P* < 0.05. Species richness and evenness of the samples were assessed by calculating the Shannon index, based on the species profile, using the phyloseq v.1.44 package in R software^45,46^. The graphs were generated using the ‘ggplot2’ R package (version 3.5.0)^47,48^, and heatmaps were generated with pheatmap R package (version 1.0.12)^49^. Venn diagrams were generated with limma R package (version 3.28.14)^50^. By using Spearman correlation, correlation plots were constructed using the corrplot R package (version 0.92)^51^.To examine the differences in microbial community structures between groups, PCoA was conducted using the vegan v.2.6-4 package in R^52^.

### In silico identification of MDRBs

Multidrug-resistant bacterial species (MDRBs) were identified in *silico* from metagenomic data by detecting species with resistance to three or more antibiotic classes.

### List of commensals, HAI species and foodborne pathogens

All the commensal species, HAI species and foodborne pathogens used for the analysis were listed in Supplementary File 2, with references for all taxa^53–98^.

## Electronic supplementary material

Below is the link to the electronic supplementary material.

Supplementary Material 1

Supplementary Material 2

Supplementary Material 3

Supplementary Material 4

Supplementary Material 5

## Data Availability

All sequence data used in the analyses were deposited in the Sequence Read Archive (SRA) (http://www.ncbi.nlm.nih.gov/sra) under PRJNA1194338. Until publication, please use reviewers link: https://dataview.ncbi.nlm.nih.gov/object/PRJNA1194338?reviewer=h0j5mrc16g674sf19tmin7ugsl.
