# Supplementary material for: Comprehensive analysis of antimicrobial resistance dynamics among broiler and duck intensive production systems
Source: Sci Rep. 2025 Feb 8;15:4673. doi: 10.1038/s41598-025-89432-z (PMC11806100; doi:10.1038/s41598-025-89432-z)
Supplement: Supplementary file 1 — Supplementary Material 1 [file 41598_2025_89432_MOESM1_ESM.docx]

| Table of short-chain fatty acid (SCFA) producers, probiotic strains, hospital-acquired infection (HAI) causing species and foodborne pathogens | | | |
| --- | --- | --- | --- |
| Species | SCFA-producer | Probiotic | SCFA-producer and probiotic |
| *Bacillus subtilis* |  | x ^[54]^ |  |
| *Bacteroides fragilis* |  | x ^[55]^ |  |
| *Bacteroides thetaiotaomicron* |  | x ^[56]^ |  |
| *Bifidobacterium adolescentis* |  |  | x ^[57]^ |
| *Bifidobacterium bifidum* |  |  | x ^[57]^ |
| *Bifidobacterium breve* |  |  | x ^[58]^ |
| *Butyrivibrio fibrisolvens* | x ^[59]^ |  |  |
| *Clostridium butyricum* |  |  | x ^[60]^ |
| *Lactobacillus johnsonii* |  |  | x ^[57]^ |
| *Lactococcus lactis* |  |  | x ^[61]^ |
| *Limosilactobacillus reuteri* |  | x ^[62]^ |  |
| *Parabacteroides distasonis* | x ^[63]^ |  |  |
| *Phocaeicola vulgatus* | x ^[64]^ |  |  |
| *Prevotella bivia* |  | x ^[65]^ |  |
| *Prevotella intermedia* |  | x ^[66]^ |  |
| List of hospital-acquired infection (HAI) causing species | | | |
| *Acinetobacter baumannii* ^[67]^  *Acinetobacter bereziniae* ^[68]^  *Acinetobacter lwoffii* ^[69]^  *Acinetobacter nosocomialis* ^[70]^  *Acinetobacter pittii* ^[70]^  *Burkholderia cepacia* ^[71]^  *Citrobacter braakii* ^[72]^  *Citrobacter freundii* ^[73]^  *Clostridioides difficile* ^[74]^  *Corynebacterium striatum* ^[75]^  *Enterobacter cloacae* ^[76]^  *Enterococcus faecalis* ^[77]^  *Enterococcus faecium* ^[76]^  *Escherichia coli* ^[78]^  *Haemophilus influenzae* ^[79]^  *Klebsiella oxytoca* ^[80]^  *Klebsiella pneumoniae* ^[68]^  *Morganella morganii* ^[81]^  *Proteus mirabilis* ^[82]^  *Pseudomonas aeruginosa* ^[67]^  *Serratia marcescens* ^[83]^  *Staphylococcus aureus* ^[68]^  *Staphylococcus epidermidis* ^[84]^  *Staphylococcus haemolyticus* ^[85]^  *Stenotrophomonas maltophilia* ^[86]^  *Streptococcus pneumoniae* ^[87]^  *Streptococcus pyogenes* ^[88]^ | | | |
| List of foodborne pathogens | | | |
| *Bacillus cereus* ^[89]^  *Campylobacter coli* ^[90]^  *Campylobacter jejuni* ^[91]^  *Clostridium botulinum* ^[92]^  *Clostridium perfringens* ^[93]^  *Helicobacter pylori* ^[94]^  *Listeria monocytogenes* ^[95]^  *Salmonella enterica* ^[96]^  *Shigella flexneri* ^[97]^  *Shigella sonnei* ^[98]^  *Yersinia enterocolitica* ^[99]^ | | | |

**References**

54. Williams, N. & Weir, T. L. Spore-Based Probiotic Bacillus subtilis: Current Applications in Humans and Future Perspectives. *Fermentation* 10, 78 (2024).

55. He, Q. *et al.* Protective effects of a new generation of probiotic Bacteroides fragilis against colitis in vivo and in vitro. *Sci. Rep.* 13, 15842 (2023).

56. Lalowski, P. & Zielińska, D. The Most Promising Next-Generation Probiotic Candidates—Impact on Human Health and Potential Application in Food Technology. *Fermentation* 10, 444 (2024).

57. Fusco, W. *et al.* Short-Chain Fatty-Acid-Producing Bacteria: Key Components of the Human Gut Microbiota. *Nutrients* 15, 2211 (2023).

58. Yoon, S. J. *et al.* Bifidobacterium-derived short-chain fatty acids and indole compounds attenuate nonalcoholic fatty liver disease by modulating gut-liver axis. *Front. Microbiol.* 14, (2023).

59. Ohkawara, S., Furuya, H., Nagashima, K., Asanuma, N. & Hino, T. Oral Administration of *Butyrivibrio fibrisolvens*, a Butyrate-Producing Bacterium, Decreases the Formation of Aberrant Crypt Foci in the Colon and Rectum of Mice12. *J. Nutr.* 135, 2878–2883 (2005).

60. Chen, D. *et al.* Clostridium butyricum, a butyrate-producing probiotic, inhibits intestinal tumor development through modulating Wnt signaling and gut microbiota. *Cancer Lett.* 469, 456–467 (2020).

61. De Chiara, I. *et al.* Probiotic Properties of Lactococcus lactis Strains Isolated from Natural Whey Starter Cultures. *Foods* 13, 957 (2024).

62. Abuqwider, J., Altamimi, M. & Mauriello, G. Limosilactobacillus reuteri in Health and Disease. *Microorganisms* 10, 522 (2022).

63. Lei, Y. *et al.* Parabacteroides produces acetate to alleviate heparanase-exacerbated acute pancreatitis through reducing neutrophil infiltration. *Microbiome* 9, 115 (2021).

64. Keitel, L., Miebach, K., Rummel, L., Yordanov, S. & Büchs, J. Process analysis of the anaerobe Phocaeicola vulgatus in a shake flasks and fermenter reveals pH and product inhibition. *Ann. Microbiol.* 74, 7 (2024).

65. Pybus, V. & Onderdonk, A. B. The Effect of pH on Growth and Succinate Production by *Prevotella bivia*. *Microb. Ecol. Health Dis.* 9, 19–25 (1996).

66. Leonov, G. E., Varaeva, Y. R., Livantsova, E. N. & Starodubova, A. V. The Complicated Relationship of Short-Chain Fatty Acids and Oral Microbiome: A Narrative Review. *Biomedicines* 11, 2749 (2023).

67. *Guidelines for the Prevention and Control of Carbapenem-Resistant Enterobacteriaceae, Acinetobacter Baumannii and Pseudomonas Aeruginosa in Health Care Facilities*. (World Health Organization, Geneva, 2017).

68. Reyes, S. M., Bolettieri, E., Allen, D. & Hay, A. G. Genome Sequences of Four Strains of Acinetobacter bereziniae Isolated from Human Milk Pumped with a Personal Breast Pump and Hand-Washed Milk Collection Supplies. *Microbiol. Resour. Announc.* (2020) doi:10.1128/mra.00770-20.

69. Regalado, N. G., Martin, G. & Antony, S. J. *Acinetobacter lwoffii*: Bacteremia associated with acute gastroenteritis. *Travel Med. Infect. Dis.* 7, 316–317 (2009).

70. Chusri, S. *et al.* Clinical Outcomes of Hospital-Acquired Infection with Acinetobacter nosocomialis and Acinetobacter pittii. *Antimicrob. Agents Chemother.* (2014) doi:10.1128/aac.02992-14.

71. Häfliger, E., Atkinson, A. & Marschall, J. Systematic review of healthcare-associated Burkholderia cepacia complex outbreaks: presentation, causes and outbreak control. *Infect. Prev. Pract.* 2, 100082 (2020).

72. Mohan, V., Golledge, C., Grasko, J. & Grasko, Y. Citrobacter braakii urinary tract infection in chronic kidney disease. *Microbiol. Aust.* 45, 151–154 (2024).

73. Liu, L.-H. *et al.* *Citrobacter freundii* bacteremia: Risk factors of mortality and prevalence of resistance genes. *J. Microbiol. Immunol. Infect.* 51, 565–572 (2018).

74. Lev, V. *et al.* Health care–associated Clostridioides difficile infection: Learning the perspectives of health care workers to build successful strategies. *Am. J. Infect. Control* 52, 284–292 (2024).

75. Daisuke, U., Oishi, T., Yamane, K. & Terada, K. Corynebacterium striatum Bacteremia Associated with a Catheter-Related Blood Stream Infection. *Case Rep. Infect. Dis.* 2017, 2682149 (2017).

76. Abban, M. K., Ayerakwa, E. A., Mosi, L. & Isawumi, A. The burden of hospital acquired infections and antimicrobial resistance. *Heliyon* 9, e20561 (2023).

77. Zaheer, R. *et al.* Surveillance of Enterococcus spp. reveals distinct species and antimicrobial resistance diversity across a One-Health continuum. *Sci. Rep.* 10, 3937 (2020).

78. Cheong, H. S. *et al.* Clinical significance of healthcare-associated infections in community-onset Escherichia coli bacteraemia. *J. Antimicrob. Chemother.* 60, 1355–1360 (2007).

79. Foxwell, A. R., Kyd, J. M. & Cripps, A. W. Nontypeable Haemophilus influenzae: Pathogenesis and Prevention. *Microbiol. Mol. Biol. Rev.* 62, 294–308 (1998).

80. Lowe, C. *et al.* Outbreak of Extended-Spectrum β-Lactamase–producing Klebsiella oxytoca Infections Associated with Contaminated Handwashing Sinks - Volume 18, Number 8—August 2012 - Emerging Infectious Diseases journal - CDC. doi:10.3201/eid1808.111268.

81. Alsaadi, A. *et al.* Epidemiology and clinical characteristics of *Morganella morganii* infections: A multicenter retrospective study. *J. Infect. Public Health* 17, 430–434 (2024).

82. Rus, M. *et al.* Association of Proteus mirabilis and Providencia stuartii Infections with Diabetes. *Medicina (Mex.)* 58, 271 (2022).

83. Hanczvikkel, A. *et al.* Nosocomial outbreak caused by disinfectant-resistant Serratia marcescens in an adult intensive care unit, Hungary, February to March 2022. *Eurosurveillance* 29, 2300492 (2024).

84. Widerström, M. Commentary: Significance of Staphylococcus epidermidis in Health Care-Associated Infections, from Contaminant to Clinically Relevant Pathogen: This Is a Wake-Up Call! *J. Clin. Microbiol.* 54, 1679–1681 (2016).

85. Rossi, C. C., Ahmad, F. & Giambiagi-deMarval, M. *Staphylococcus haemolyticus:* An updated review on nosocomial infections, antimicrobial resistance, virulence, genetic traits, and strategies for combating this emerging opportunistic pathogen. *Microbiol. Res.* 282, 127652 (2024).

86. Keaton, A., Fike, L., Spicer, K., Kallen, A. & Perkins, K. Healthcare-associated Stenotrophomonas maltophilia infections in the United States, 2018–2022. *Antimicrob. Steward. Healthc. Epidemiol. ASHE* 3, s89 (2023).

87. Cedrone, F. *et al.* The Burden of Streptococcus pneumoniae-Related Admissions and In-Hospital Mortality: A Retrospective Observational Study between the Years 2015 and 2022 from a Southern Italian Province. *Vaccines* 11, 1324 (2023).

88. Avire, N. J., Whiley, H. & Ross, K. A Review of Streptococcus pyogenes: Public Health Risk Factors, Prevention and Control. *Pathogens* 10, 248 (2021).

89. Bottone, E. J. Bacillus cereus, a Volatile Human Pathogen. *Clin. Microbiol. Rev.* 23, 382–398 (2010).

90. Tam, C. C., O’Brien, S. J., Adak, G. K., Meakins, S. M. & Frost, J. A. *Campylobacter coli*—an important foodborne pathogen. *J. Infect.* 47, 28–32 (2003).

91. Altekruse, S. F., Stern, N. J., Fields, P. I. & Swerdlow, D. L. Campylobacter jejuni—An Emerging Foodborne Pathogen - Volume 5, Number 1—February 1999 - Emerging Infectious Diseases journal - CDC. doi:10.3201/eid0501.990104.

92. Rawson, A. M., Dempster, A. W., Humphreys, C. M. & Minton, N. P. Pathogenicity and virulence of Clostridium botulinum. *Virulence* 14, 2205251.

93. García, S. & Heredia, N. Clostridium perfringens: A Dynamic Foodborne Pathogen. *Food Bioprocess Technol.* 4, 624–630 (2011).

94. Quaglia, N. C. & Dambrosio, A. Helicobacter pylori: A foodborne pathogen? *World J. Gastroenterol.* 24, 3472–3487 (2018).

95. Farber, J. M. & Peterkin, P. I. Listeria monocytogenes, a food-borne pathogen. *Microbiol. Rev.* 55, 476–511 (1991).

96. Fàbrega, A. & Vila, J. Salmonella enterica Serovar Typhimurium Skills To Succeed in the Host: Virulence and Regulation. *Clin. Microbiol. Rev.* 26, 308–341 (2013).

97. Zaika, L. L., Phillips, J. G., Fanelli, J. S. & Scullen, O. J. Revised model for aerobic growth of *Shigella flexneri* to extend the validity of predictions at temperatures between 10 and 19°C. *Int. J. Food Microbiol.* 41, 9–19 (1998).

98. Matanza, X. M. & Clements, A. Pathogenicity and virulence of Shigella sonnei: A highly drug-resistant pathogen of increasing prevalence. *Virulence* 14, 2280838.

99. Mancini, M. E. *et al.* Isolation and characterization of Yersinia enterocolitica from foods in Apulia and Basilicata regions (Italy) by conventional and modern methods. *PLoS ONE* 17, e0268706 (2022).
